# Paleoamerican exploitation of extinct megafauna revealed through immunological blood residue and microwear analysis, North and South Carolina, USA

**DOI:** 10.1038/s41598-023-36617-z

**Published:** 2023-06-10

**Authors:** Christopher R. Moore, Larry R. Kimball, Albert C. Goodyear, Mark J. Brooks, I. Randolph Daniel, Allen West, Sean G. Taylor, Kiersten J. Weber, John L. Fagan, Cam M. Walker

**Affiliations:** 1 South Carolina Department of Natural Resources (SCDNR), Land, Water and Conservation Division, Heritage Trust Program, PO Box 167, Columbia, SC 29202 USA; 2 South Carolina Institute for Archaeology and Anthropology, University of South Carolina, 1321 Pendleton Street, Columbia, SC 29208 USA; 3 Department of Anthropology, Appalachian State University, Boone, NC 28608 USA; 4 Department of Anthropology, East Carolina University, Greenville, NC 27858 USA; 5 Comet Research Group, Prescott, AZ USA; 6 Archaeological Investigations Northwest, 3510 N.E. 122nd Ave., Portland, OR 97230 USA; 7 WWAMI Medical Education Program, University of Wyoming, Dept. 4238 Health Sciences Bldg., Rm. 445E, 1000 E. University Avenue, Laramie, WY 82071 USA

**Keywords:** Anthropology, Archaeology, Palaeontology

## Abstract

Previous immunological studies in the eastern USA have failed to establish a direct connection between Paleoamericans and extinct megafauna species. The lack of physical evidence for the presence of extinct megafauna begs the question, did early Paleoamericans regularly hunt or scavenge these animals, or were some megafauna already extinct? In this study of 120 Paleoamerican stone tools from across North and South Carolina, we investigate this question using crossover immunoelectrophoresis (CIEP). We find immunological support for the exploitation of extant and extinct megafauna, including Proboscidea, Equidae, and Bovidae (possibly *Bison antiquus*), on Clovis points and scrapers, as well as possible early Paleoamerican Haw River points. Post-Clovis points tested positive for Equidae and Bovidae but not Proboscidea. Microwear results are consistent with projectile usage, butchery, fresh- and dry hide scraping, the use of ochre-coated dry hides for hafting, and dry hide sheath wear. This study represents the first direct evidence of the exploitation of extinct megafauna by Clovis and other Paleoamerican cultures in the Carolinas and more broadly, across the eastern United States, where there is generally poor to non-existent faunal preservation. Future CIEP analysis of stone tools may provide evidence on the timing and demography of megafaunal collapse leading to eventual extinction.

## Introduction

Numerous immunological studies of prehistoric chipped stone tools have provided evidence consistent with the preservation of prehistoric blood protein residues (Williamson et al.^1^; Downs and Lowenstein^2^; Gerlach et al*.*^3^; Hardy et al*.*^4^; Hyland et al*.*^5^; Kooyman et al*.*^6^; Kooyman et al*.*^7^; Lowenstein^8,9^; Loy and Dixon^10^; Newman^11^; Newman and Julig^12^; Newman et al*.*^13^; Moore et al*.*^14^; Shanks et al*.*^15^; Gill-King^16^; Seeman et al*.*^17^; Yohe and Bamforth^18^; Nowell et al*.*^19^). These studies have provided valuable insight into prehistoric human/animal interactions with consistent ecological implications derived from the archaeological record (i.e. concordance between preserved faunal remains and immunological results). For example, Moore et al*.*^14^ recovered large numbers of gastroliths and calcined fragments of avian bone from Flamingo Bay (38AK469) indicating extensive processing of large birds. Immunological testing using crossover immunoelectrophoresis (CIEP) subsequently identified turkey along with quail, grouse, or other gallinaceous fowl on stone tools from the site. Despite this, CIEP has not been without skeptics.

In a previous publication (Moore et al*.*^14^), several of the co-authors of this current paper cited the work of Shanks et al.^15^ and noted that:

“Several studies have cast doubt on the reliability and accuracy of CIEP results, with skepticism concerning the survivability of animal proteins for long periods and the ability of CIEP to identify those residues (e.g. Fiedel^20^; Grayson and Meltzer^21^; Vance^22^). This criticism notwithstanding, proteins recovered on archaeological and experimental stone tools have been found to be tenacious (Shanks et al^23^), with protein derivatives preserved within stone microfractures as linear epitopes (Abbas et al.^24^; Sensabaugh et al.^25,26^; Shanks et al.^15^). Experimental studies show that microfractures produced during stone tool manufacture rapidly absorb proteins due to capillary uptake during tool use (Shanks et al.^15^). The absorption of proteins below the surface of the artifact likely acts to protect and preserve proteins preventing their removal during routine washing of artifacts after recovery and may explain how proteins can be identified by immunological testing of heavily weathered stone tools. Other debris and residue films may also protect more deeply embedded proteins by filling in and covering microfractures (Shanks et al.^15^). Thus, proteins may be preserved within lithic implements even in regions where acidic sandy soils preclude the likelihood of faunal preservation” (Moore et al.^14^).

In addition, Nowell et al*.*^19^ state that “*…the combination of proteins, fatty tissues, and soil particles (as would accumulate on an artifact used in hide scraping, *etc*.) is resistant to microbes. It is nearly insoluble as well, particularly if the fatty tissues have been modified by taphonomic processes into adipocere and have taken on calcium ions from either water or soil with high mineral content (Gill-King*^16^*). Experimental testing on stone tools confirmed this observation”.*

Given the absence of evidence for extinct megafauna in previous Southeastern immunological studies (e.g., Goodwin et al*.*^27^; McAvoy and McAvoy^28,29^; Moore et al*.*^14^), and the relatively small percentage of artifacts that typically produce reactions to antisera, the purpose of this study is to examine a much larger sample of Paleoamerican bifaces and scrapers (n = 120) to determine if blood protein residues from extinct megafauna are present. In this study, we collected 120 Paleoamerican stone tools gathered from across North and South Carolina and used crossover immunoelectrophoresis (CIEP) to test for Proboscidea, Equidae, Camelidae, and Bovidae. Additional immunological testing was performed for extant animals, including Cervidae, Canidae, Leporidae, Felidae, and Ursidae.

The lack of blood residue evidence for extinct megafauna from earlier Southeastern studies is perplexing but may simply be related to the limited number of artifacts tested. If it can be determined through CIEP that extinct megafauna were present and hunted/scavenged by Paleoamerican hunter-gatherers in the Carolinas, there are potentially significant implications for understanding early human/animal interactions in a region of the U.S. that typically displays minimal bone preservation in terrestrial environments. The identification of extinct megafauna blood residues also has implications for the timing of megafaunal extinctions in the region, the ecological conditions related to the presence or absence of these animals on the landscape, and our presumptions about the availability and the relative importance of these species in the diet of various Paleoamerican cultures (Faith and Surovell^30^; Gill et al*.*^31^; Russell et al*.*^32^).

## Artifacts

**Figure 1:**
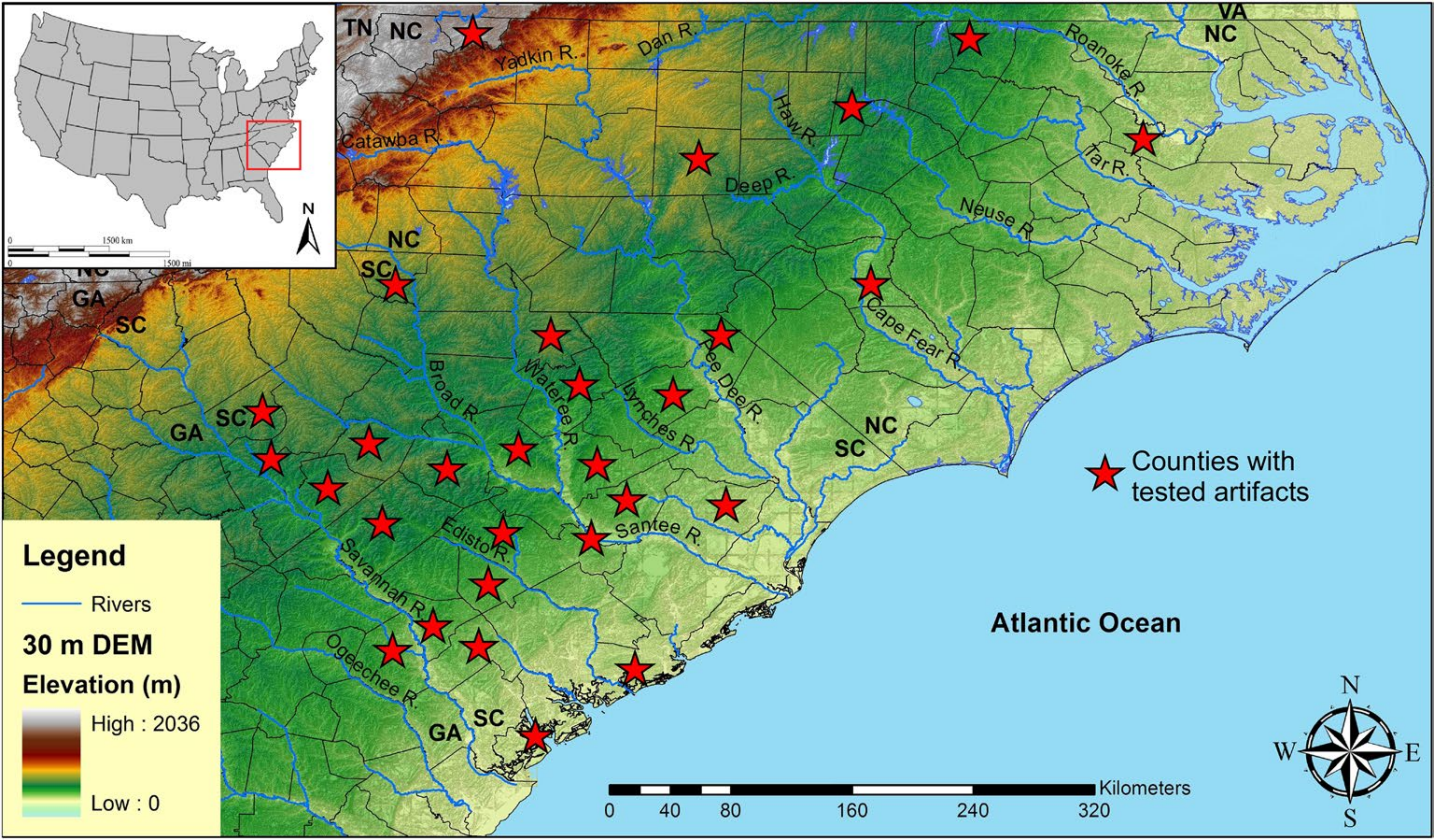
Locations of tested artifacts by state and by county using CIEP. 30-m digital elevation map (DEM) produced in ArcGIS software (v.10.4.1).

Paleoamerican artifacts (n = 120) gathered from across North and South Carolina included artifacts held within local county museums, military installations, universities, and private collections. Among these artifacts are 71 Clovis points, two unifacial scrapers, 31 early Paleoamerican Haw River points, 11 full-fluted Redstone points, two Simpson points, one Quad point, one Cumberland, and one Beaver Lake (Fig. 1; Supplementary Table 2). The vast majority of these artifacts are from surface context; however, one Clovis and two associated unifacial scrapers were excavated together as part of an archaeological mitigation project in a deeply buried alluvial context at 38LX531 (Tree House Site) on the Saluda River in South Carolina (Nagle and Green^33^: Fig. 9.1). The Clovis artifacts from 38LX531 were found at a depth of more than 2 m in an alluvial sequence containing well-stratified Woodland through Paleoamerican occupations (Nagle and Green^33^). A Redstone point fragment was also excavated from the Sandstone Ledge Rockshelter (38LX283) in South Carolina (Steen and Judge^34^) (Supplementary Table 2).

In all cases, Paleoamerican artifacts were previously held/inspected and some but not all were washed and labeled before the undertaking of this study. The extremely limited occurrence of buried Paleoamerican sites in the Carolinas prohibited the use of any “pristine” or unhandled/unwashed artifacts in this study. The authors acknowledge that post-depositional contamination is possible, but we argue that is an unlikely source of animal proteins detected with CIEP. This is because blood protein residues from prehistoric animal species are preserved as linear epitopes within stone microfractures that are sealed with the accumulation of sediments and lipids during use of the artifact in butchery or hunting activities (Shanks et al*.*^15^; Nowell et al*.*^19^). More recent animal proteins that might be encountered in sediments in the form of urine or feces are unlikely to be absorbed within microfractures that have long since been filled. Moreover, modern animal proteins within urine or feces degrade rapidly on the surface of artifacts or within surface sediments. There are a number of studies that assert that the survival interval of immunoglobulin proteins on or in soil is less than two years, even under ideal circumstances (Nowell et al*.*^19^). Additionally, non-circulating bodily fluids have immunoglobulin concentrations that are orders of magnitude less than other fluids, such as blood, lymph, and interstitial fluid [such as in adipose tissue].

Urine, in particular, should have extremely low immunoglobulin content [near, if not at zero], as kidney function is designed to retain blood cells and proteins within our blood composition, not excrete them (Williamson et al*.*^1^).

## Results and discussion

**Figure 2:**
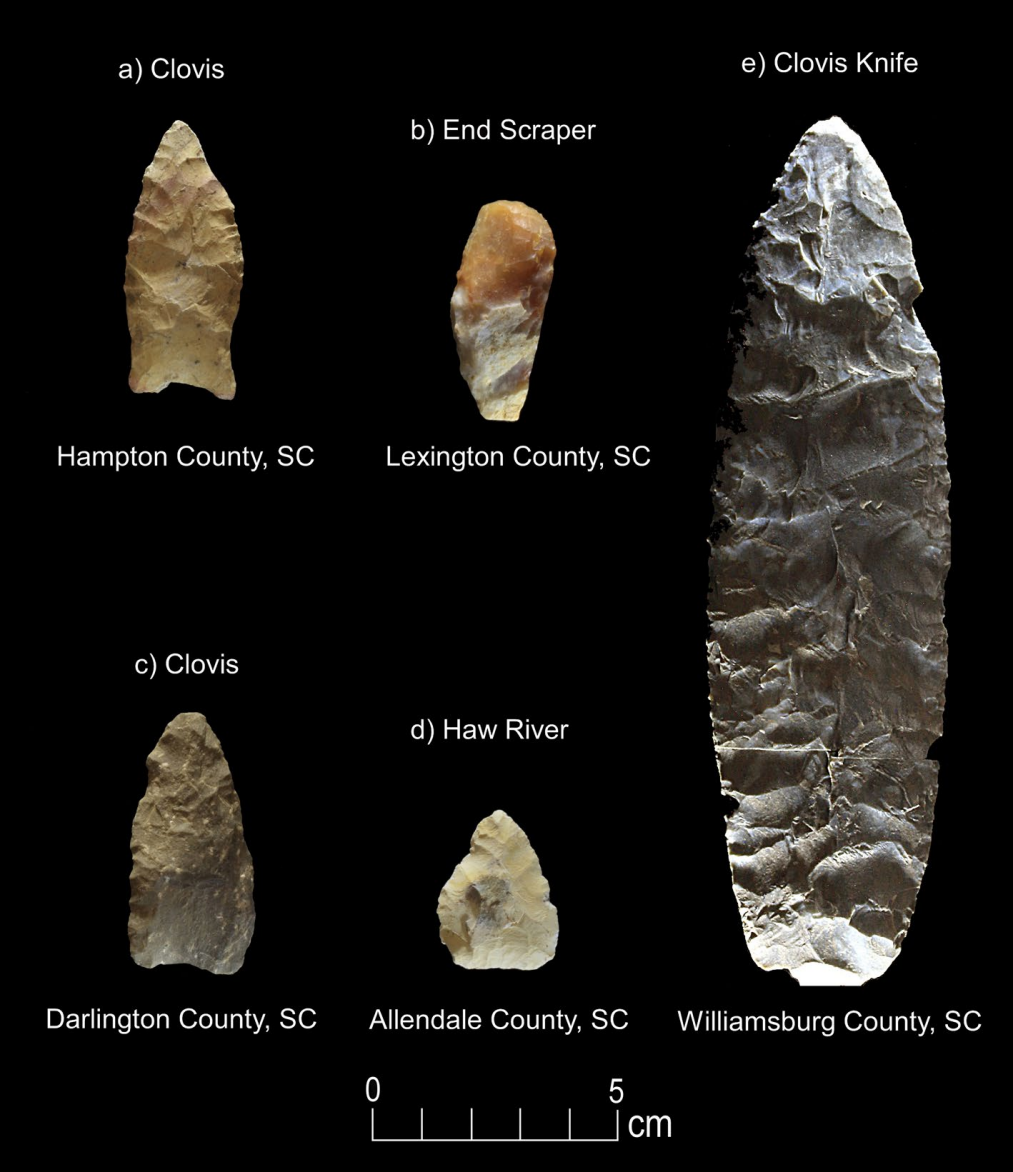
Paleoamerican artifacts positive for Proboscidea: (**a**) Clovis point from Hampton County, South Carolina (#1), (**b**) Clovis end scraper from Lexington County, South Carolina (#29), (**c**) Clovis from Darlington County, South Carolina (#66), (**d**) possible early Paleoamerican Haw River biface (#117), and (**e**) large Clovis knife from Williamsburg County, South Carolina (#120) (Table 1).

**Figure 3:**
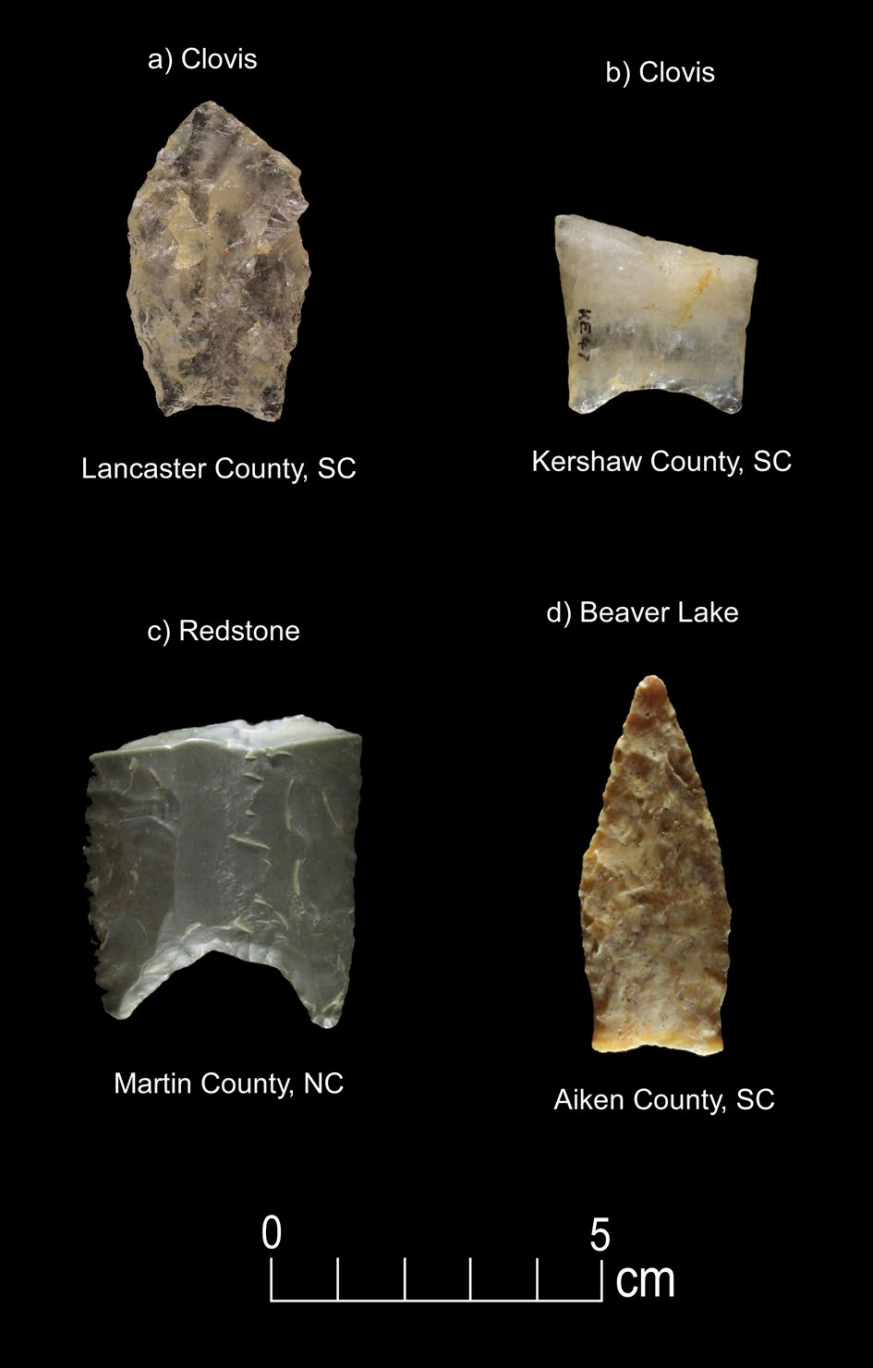
Paleoamerican artifacts positive for Equidae: (**a**) Clovis point from Lancaster County, South Carolina (#101), (**b**) Clovis base from Kershaw County, South Carolina (#94), (**c**) Redstone from Martin County, North Carolina (#93), and (**d**) Beaver Lake point from Aiken County, South Carolina (#85) (Table 1).

**Figure 4:**
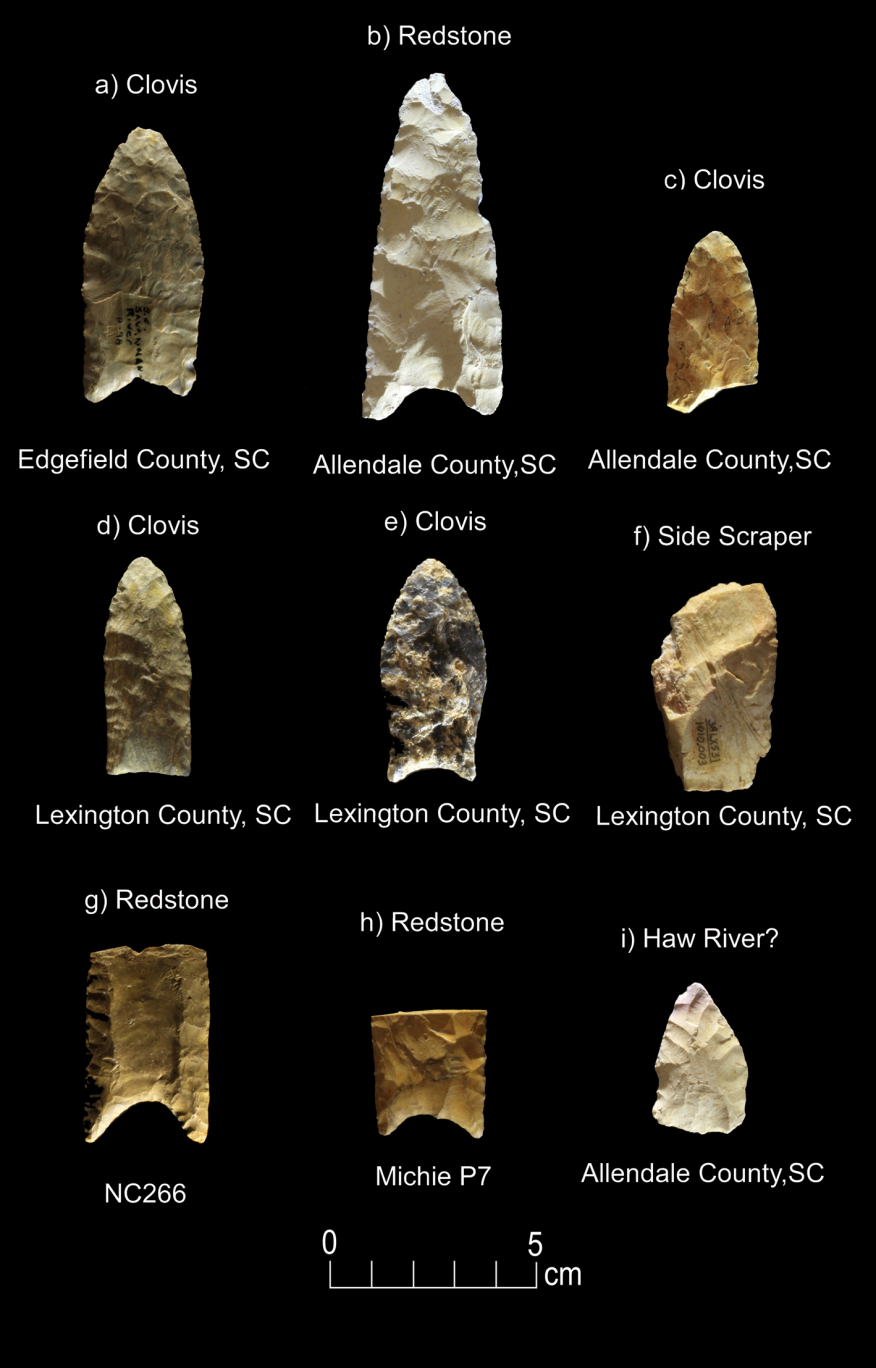
Paleoamerican artifacts positive for Bovidae: (**a**) Clovis point from Edgefield County, South Carolina (#54), (**b**) Redstone point from Allendale County, South Carolina (#38), (**c**) Clovis from Lexington County, South Carolina (#37), (**d**) Clovis from Lexington County, South Carolina (#61), (**e**) Clovis from Lexington County, South Carolina (#27), (**f**) Clovis Side Scraper from Lexington County, South Carolina (#28), (**g**) Redstone from North Carolina (#74), (**h**) Redstone base from South Carolina (#46), and possible early Paleoamerican Haw River point from Allendale County, South Carolina (#114) (Table 1).

**Figure 5:**
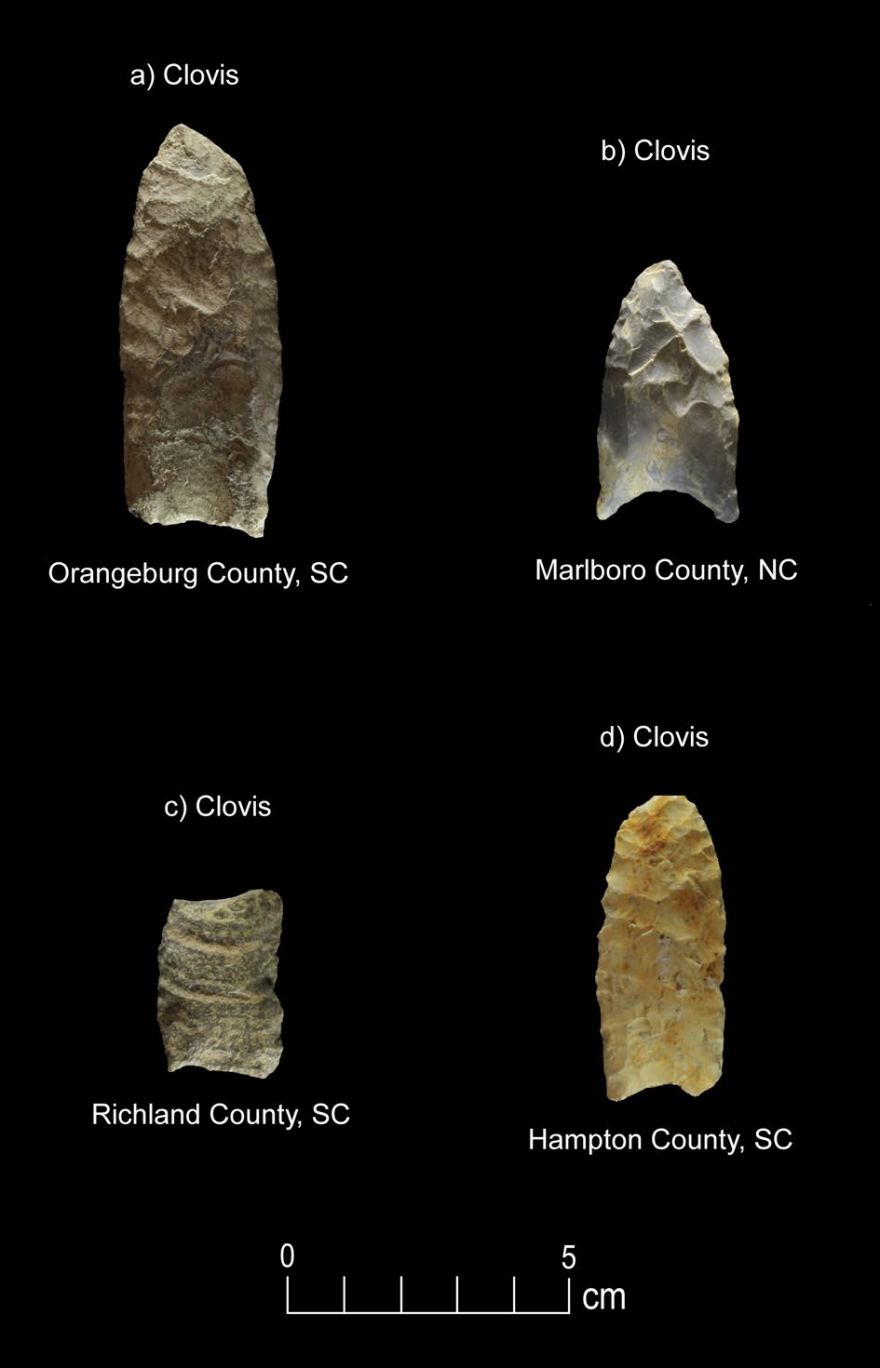
Paleoamerican artifacts positive for Cervidae: (**a**) Clovis point from Orangeburg County, South Carolina (#59), (**b**) Clovis point from Marlboro County, South Carolina (#52), (**c**) Clovis from Richland County, South Carolina (#80), (**d**) Clovis from Hampton County, South Carolina (#4) (Table 1).

**Figure 6:**
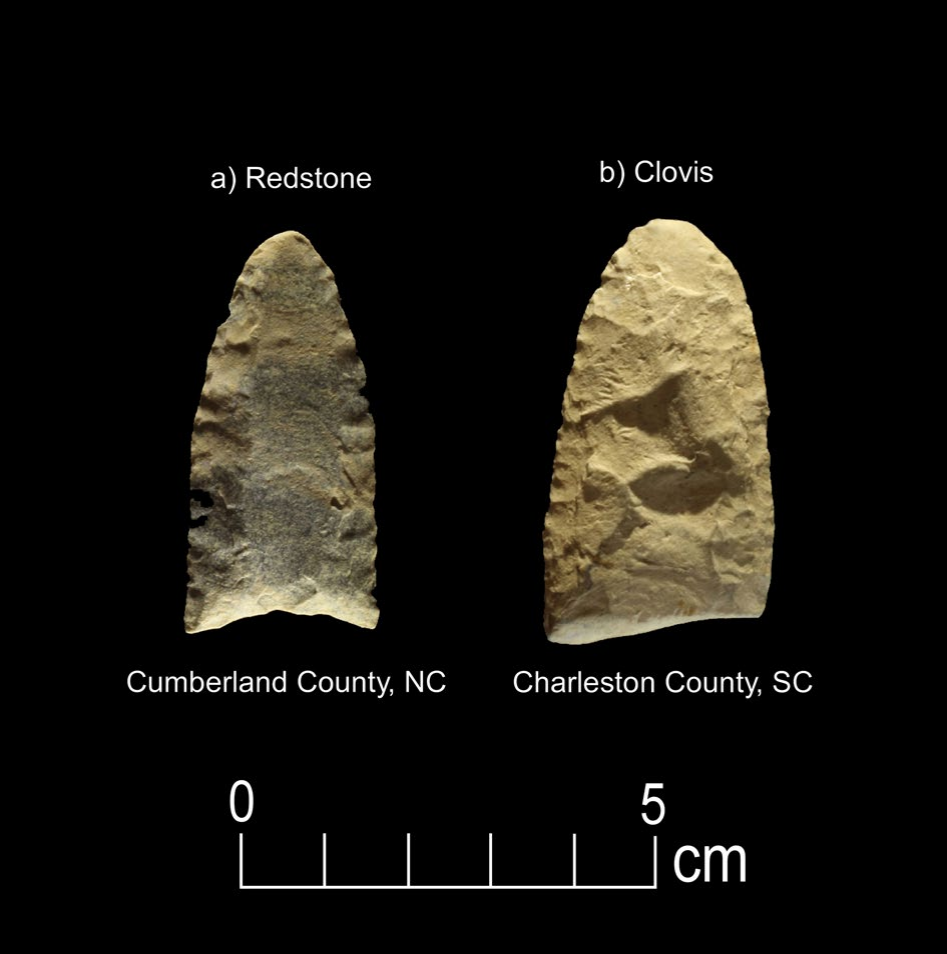
Paleoamerican artifacts positive for Canidae: (**a**) Redstone point from Cumberland County, North Carolina (#106), and (**b**) Clovis point from Charleston County, South Carolina (#45) (Table 1).

**Figure 7:**
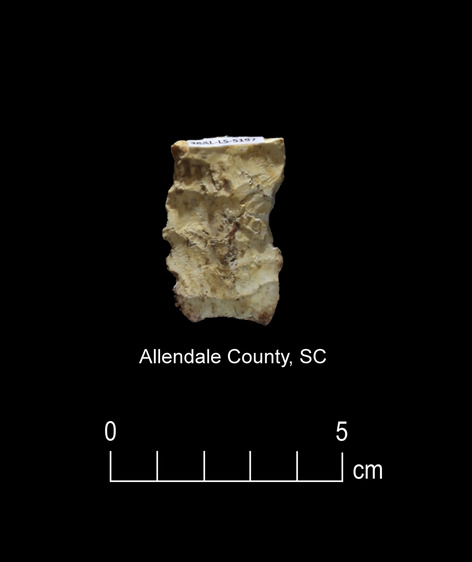
Paleoamerican artifact positive for Ursidae: Possible early Paleoamerican Haw River point from Allendale County, South Carolina (#109) (Table 1).

**Figure 8:**
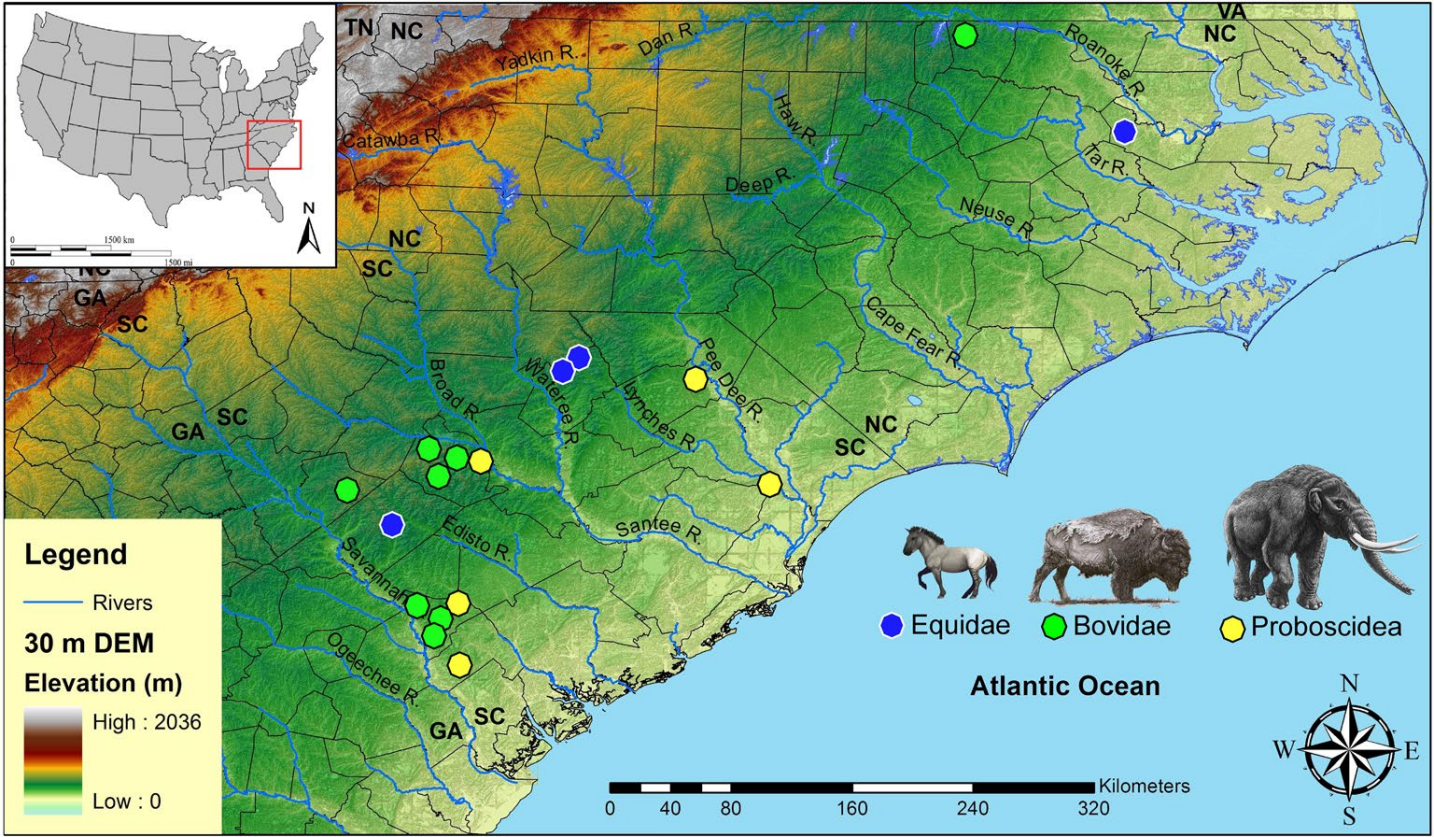
Locations by state and by county of CIEP tested artifacts and positive reactions for Equidae, Bovidae, and Proboscidea (Table 1). One artifact (#46; positive for Bovidae) is from South Carolina but has no precise provenience and is not plotted on the figure. 30-m digital elevation map (DEM) produced in ArcGIS software (v.10.4.1). The Equidae (Pleistocene horse) image is used with permission of Benji Paysnoe and is owned by the National Parks Service (NPS) and is in the public domain. The Bovidae image (American Bison) by Chris Woolley is reproduced with permission of Connie Woolley and the American Mastodon image is courtesy of La Brea Tar Pits, Los Angeles County Museum of Natural History.

**Figure 9:**
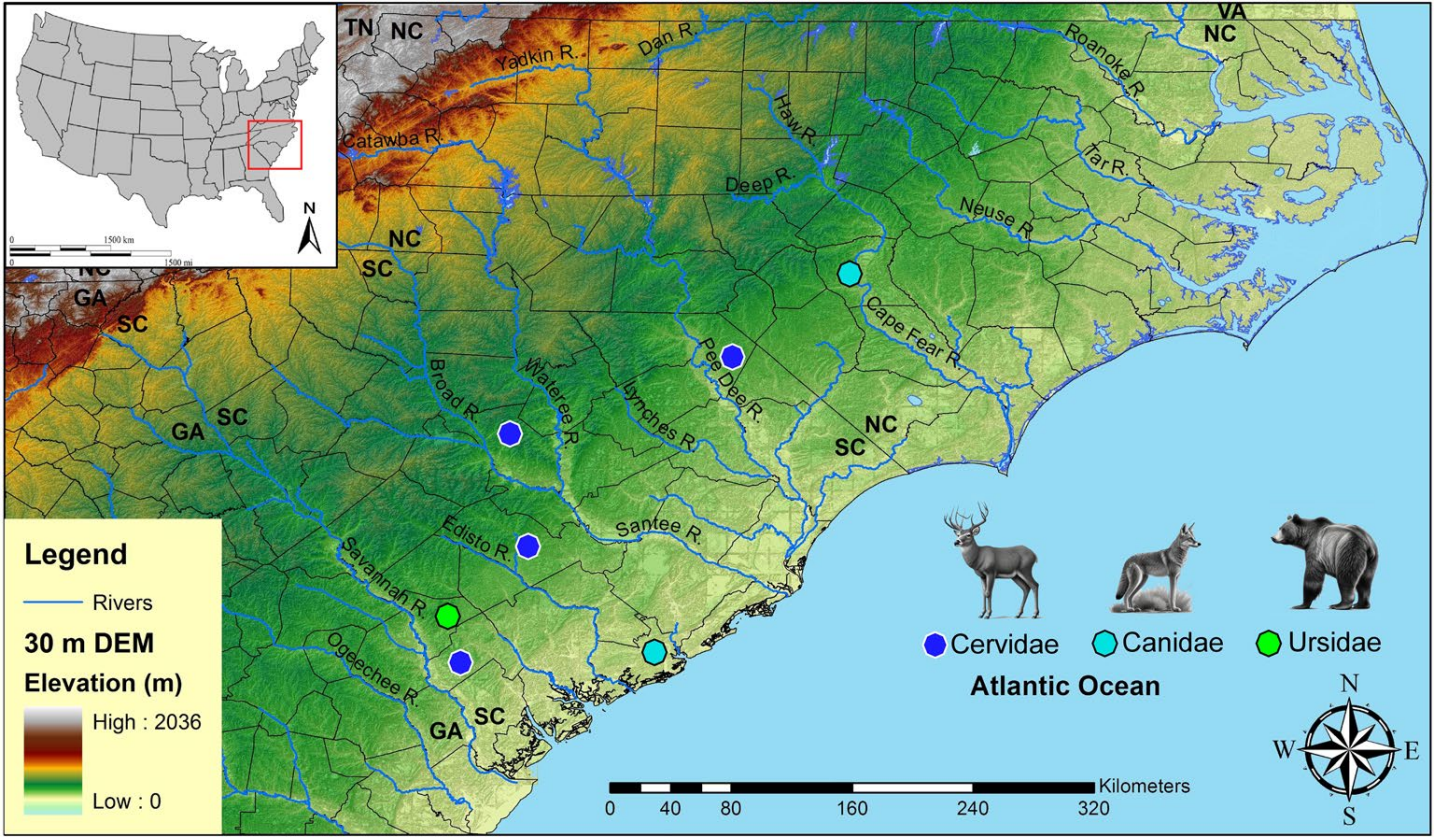
Locations by state and by county of CIEP tested artifacts and positive reactions for Cervidae, Canidae, and Ursidae (Table 1). 30-m digital elevation map (DEM) produced in ArcGIS software (v.10.4.1). Animal images produced with AI software by Midjourney^©^.

*CIEP.* Results of crossover immunoelectrophoresis (CIEP) analysis on 120 Paleoamerican stone tools included testing for extinct and extant animals (Figs. 2, 3, 4, 5, 6, 7, 8, 9 and Table 1). Extinct species include Proboscidea, Equidae, and Bovidae, which produced 18 positive reactions, or 15% out of the 120 artifacts tested against available antisera. These included five positive reactions to Proboscidea (four Clovis artifacts and one possible early Paleoamerican Haw River point), four positive reactions to Equidae (two Clovis, one Redstone, and one likely Clovis variant), and nine positive reactions to Bovidae (four Clovis points, one Clovis side-scraper, three Redstone points, and one possible early Paleoamerican “Haw River” point) (Figs. 2, 3, 4, 8; Table 1). A list of artifacts that tested negative for blood residue is given in Supplementary Information (Supplementary Table 3).

**Table 1:** Paleoamerican artifacts positive for blood residue.

Analysis ID	Artifact ID	County/State	Context	Type	Cultural affiliation	^1^Raw material	CIEP results
1	JC 8	Hampton/SC	Surface	Clovis	Early Paleoindian	CPC	Proboscidea
4	JC 415	Hampton/SC	Surface	Clovis	Early Paleoindian	CPC	Cervidae
27	^2^38LX531a	Lexington/SC	Excavated	Clovis	Early Paleoindian	BMC	Bovidae
28	^2^38LX531b	Lexington/SC	Excavated	Clovis Side Scraper	Early Paleoindian	Ch	Bovidae
29	^2^38LX531c	Lexington/SC	Excavated	Clovis End Scraper	Early Paleoindian	Ch	Proboscidea
37	SC766	Allendale/SC	Surface	Clovis	Early Paleoindian	CPC	Bovidae
38	SC215	Allendale/SC	Surface	Redstone	Middle Paleoindian	CPC	Bovidae
45	SC445	Charleston/SC	Surface	Clovis	Early Paleoindian	MTV	Canidae
46	P-7	SC	Surface	Redstone	Middle Paleoindian	CPC	Bovidae
52	SC641	Marlboro/SC	Surface	Clovis	Early Paleoindian	MTV	Cervidae
54	SC444	Edgefield/SC	Surface	Clovis	Early Paleoindian	Ch	Bovidae
59	SC440	Orangeburg/ SC	Surface	Clovis	Early Paleoindian	FBR	Cervidae
61	S452	Lexington/SC	Surface	Clovis	Early Paleoindian	FBR	Bovidae
66	AB#3	Darlington/SC	Surface	Clovis	Early Paleoindian	PPR	Proboscidea
74	NC266	Northhampton/NC	Surface	Redstone	Middle Paleoindian	Jas	Bovidae
80	^3^38RD18	Richland/SC	Surface	Clovis	Early Paleoindian	FBR	Cervidae
85	Arena1	Aiken/SC	Surface	Beaver Lake	Middle Paleoindian	CPC	Horse
93	NC303	Martin/NC	Surface	Redstone	Middle Paleoindian	Rhy	Horse
94	VS1	Kershaw/SC	Surface	Clovis	Early Paleoindian	Q	Horse
101	VS8	Lancaster/SC	Surface	Clovis	Early Paleoindian	CQ	Horse
106	JL1	Cumberland/NC	Surface	Redstone	Middle Paleoindian	MTV	Canidae
109	LS 5197	Allendale/SC	Surface	Haw River	Early Paleoindian	CPC	Bear
114	LS 5207	Allendale/SC	Surface	Haw River	Early Paleoindian	CPC	Bovidae
117	LS 5199	Allendale/SC	Surface	Haw River	Early Paleoindian	CPC	Proboscidea
120	BZ1	Williamsburg/SC	Surface	Clovis Knife	Early Paleoindian	Ch	Proboscidea

^1^Raw Materials types include Coastal Plain Chert (CPC), Chert (Ch), Metavolcanic (MTV), Rhyolite (Rhy), Flow-Banded Rhyolite (FBR), Aphyric Rhyolite (AR), Plagioclase-Porphyritic Rhyolite (PPR), Quartz (Q), Crystal Quartz, (CQ), Green Vitric Tuff (GVT), Black Vitric Tuff (BVT), Jasper (Jas), and Black Mingo Chert (BMC).

^2^38LX531 (Tree House Site) was excavated as part of a CRM mitigation project (Nagle and Green^33^).

^3^38RD18 (Nipper Creek Site).

The low percentage of artifacts positive for blood residue is likely because the vast majority of artifacts tested were found at or near the surface where they have been exposed to millennia of weathering. This is supported by the fact that 3 deeply buried Clovis artifacts from an archaeological mitigation at the Tree House Site (36LX531) in South Carolina all produced positive reactions to antiserum (Table 1). By comparison, 26% of the excavated Paleoamerican and Early Archaic artifacts at Flamingo Bay (38AK469) on the Savannah River Site (SRS) in South Carolina, were positive for available antiserum (Moore et al.^14^). At the La Prele mammoth site (Mackie et al*.*^35^) in Wyoming, nearly 70% of excavated artifacts tested produced positive immunological reactions, while at Hell Gap, only around 15% of the excavated artifacts were positive (Shimek et al*.*^36^). Overall, these studies support better preservation of blood residues on buried artifacts, but with exceptions to the rule that may be due to variations in soil chemistry or even differences in the fracture properties of lithic raw material.

Immunological testing for extant animals included Cervidae, Canidae, Leporidae, Felidae, and Ursidae. This analysis produced seven positive reactions (6% out of 120 artifacts tested) including four for Cervidae (four Clovis points), two for Canidae (one Redstone and one Clovis point), and one for Ursidae (one possible early Paleoamerican Haw River point) (Figs. 5, 6, 7 and 9, Table 1).

*Microwear.* Seven tools from the study sample positive for blood residue were selected for high-power microwear analysis using the Keeley method (see Kimball^37^; Moore et al*.*^14^). These tools were also selected as they were of higher quality chert or jasper, and thus, provide the best opportunity for detecting microwear traces. Specific tool morphologies include three Clovis points, one Redstone point, one end scraper, one side scraper, and one large Clovis knife. The animal residues are Proboscidea (Clovis Knife-120 and End Scraper-29); Bovidae (Clovis Points-27, 54, Redstone Point-74 and Side Scraper-28); and Horse (Clovis Point-101). Hafting traces were documented for all seven of the tools—additional proof that they were used by the tool-makers. Table 2 gives the results of the microwear analysis.

**Table 2:** Microwear results for a sample of artifacts (n = 7) positive for blood residue.

Analysis ID	Artifact ID	County/State	Type	Cultural affiliation	^1^Raw material	CIEP results	Projection?	Hafted?	Function(s)
27	38LX531a	Lexington/SC	Clovis	Early Paleoindian	BMC	Bovidae	Projection impact	Yes	Fresh hide cutting/butchery
28	38LX531b	Lexington/SC	Clovis Side Scraper	Early Paleoindian	Ch	Bovidae	–	Probable	Meat cutting
29	38LX531c	Lexington/SC	Clovis End Scraper	Early Paleoindian	Ch	Proboscidea	–	yes	Dry- hide scraping
54	SC444	Edgefield/SC	Clovis	Early Paleoindian	Ch	Bovidae	Projection impact (2)	Yes	Butchery
74	NC266	Northhampton/NC	Redstone	Middle Paleoindian	Jas	Bovidae	distal end broken	Yes	Butchery
101	VS8	Lancaster/SC	Clovis	Early Paleoindian	CQ	Horse	Distal end broken	Yes	Cutting/sawing, hide scraping
120	BZ1	Williamsburg/SC	Clovis Knife	Early Paleoindian	Ch	Proboscidea	Micro-impact fractures (2)	Yes	Butchery

^1^Raw Materials types include Chert (Ch), Crystal Quartz, (CQ), Jasper (Jas), and Black Mingo Chert (BMC).

**Figure 10:**
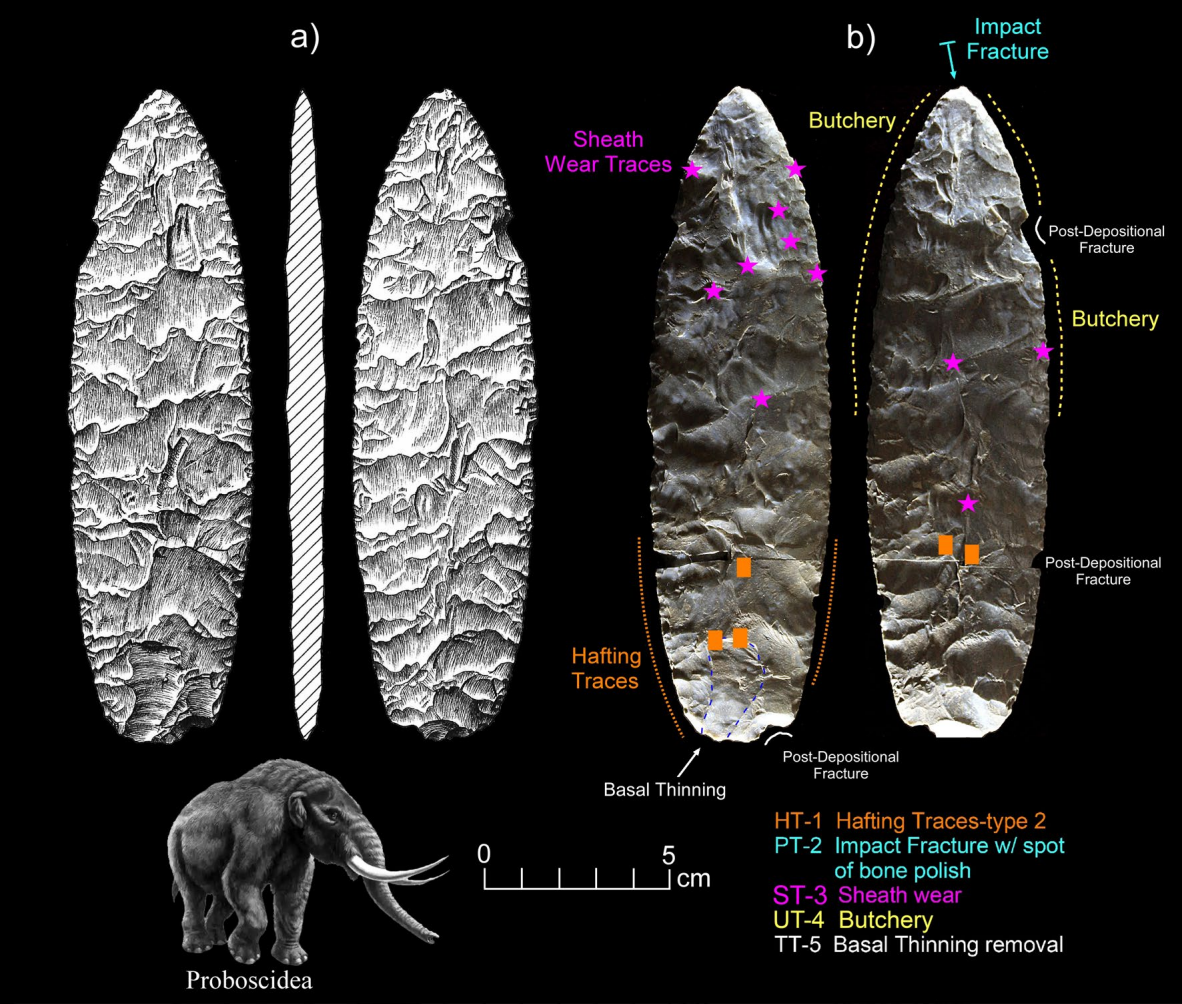
Microwear analysis for artifact #120 (Table 2) showing locations of hafting traces (HT-1), micro-impact fracture with bone polish (PT-2), sheath wear (ST-3), basal thinning (TT-5), post-depositional fractures, butchery (UT-4), and positive CIEP result (Tables 1 and 2). The American Mastodon image is courtesy of La Brea Tar Pits, Los Angeles County Museum of Natural History. Biface drawing is by Darby Erd.

**Figure 11:**
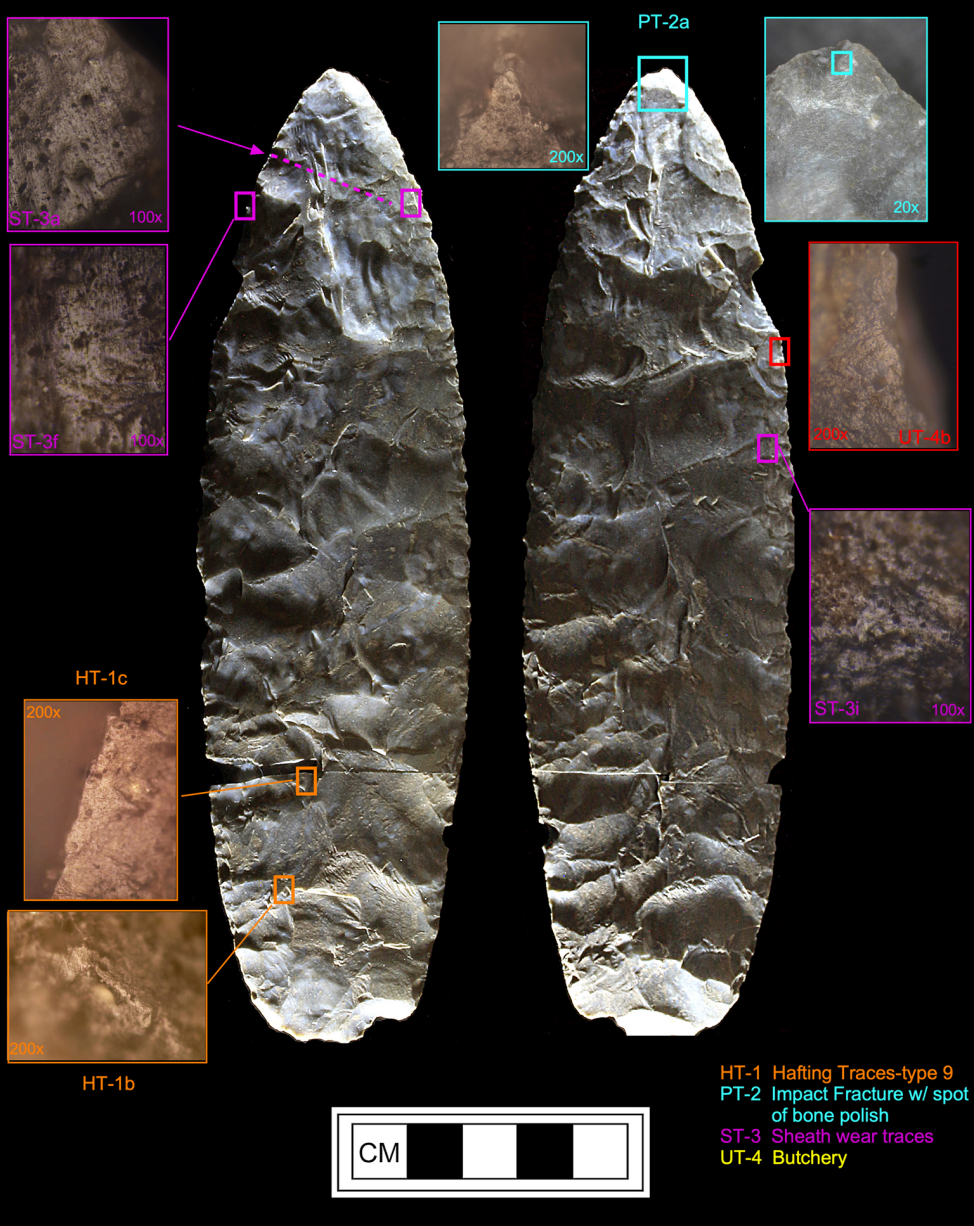
Photomicrographs for artifact #120 (Table 2) showing hafting traces (HT-1), impact fracture with bone polish (PT-2), sheath wear traces (ST-3), and butchery traces (UT-4).

Clovis Knife-120 (Fig. 10) exhibited a most interesting tool biography. This large (17.5 cm) basally thinned (TT-2) bifacial knife was made of a probable exotic chert and after it was discarded by the user, it was broken into two separated pieces, which were almost miraculously found less than one year apart in a surface context. A Clovis affiliation for this biface is based on the presence of overshot flake scars on both faces which is diagnostic of Clovis biface manufacturing. There is an attempt at end thinning or fluting on one side and there appears to be a platform set up for the reverse face. It could have been further retouched to flute the other side or simply used as is as a hafted knife which is indicated by the use-wear analysis. The presence of Proboscidea blood residue on this tool is consistent with a Clovis affiliation. The knife exhibited clear hafting traces (Fig. 11) and micro-impact fractures on both aspects of the distal tip. In addition, there is a spot of bone polish within one impact fracture (PT-2a). While bone polish may be the result of a projectile point impacting bone during impact, it has been documented experimentally (Kimball^37^: Fig. 3) that this same pattern can be the result of butchery—in that case, by hitting the sternum of a deer during experimental butchery. Both lateral edges exhibit well-expressed use-traces from butchery (UT-4).

However, different types of microtraces (Fig. 11; Supplementary Fig. 1a,b) are observed on both aspects (ST-3) which are very well-developed and unlike that expected for butchery. These microtraces are most similar to those created by scraping dry hide (Supplementary Fig. 1c). The distribution of these microtraces along the distal, lateral, and medial portions of the knife suggests a different origin. Kimball^38^ (Plate 1f) observed a similar distributional pattern on a large Dalton point/knife from 11PK1771 in western Illinois. More recently, van Gijn^39^:Fig. 7.11; van Gijn^40^:Fig. 6.4 documented strikingly similar microtraces (Supplementary Fig. 1d,e) on both experimental and archaeological daggers (Scandinavian type III Neolithic daggers). The similarity between these "sheath" polishes and their patterned distribution is remarkable (Supplementary Fig. 1d,e). However, the "sheath" polishes from Clovis Knife-120 are somewhat different, which we hypothesize is due to the Clovis-120 sheath being made of dried hide, which may have had fine sediment particles or ochre embedded in it.

There is considerable evidence that Clovis and Redstone points (Figs. 13, 14 and 15) were primarily used as projectiles and secondarily used as butchery tools, as observed on Clovis points from Williamson, VA (Kimball^41^), Flamingo Bay, SC (Kimball^42^), Birckhead, NC (Whyte and Kimball^43^); as well as at least one of the Clovis points analyzed from Gault, TX (Smallwood^44^), among others. If one can accept that projectile usage followed by butchery is a first cycle in Clovis tool biographies, then intentional or unintentional snap fractures to create scraping or planing edges could be viewed as a secondary cycle, which is documented at Williamson and Flamingo Bay. That is, one may consider a general Clovis point tool biography with two general cycles: (1) points function initially as projectiles and butchery tools; and (2) then points are modified varyingly (snap-fractures or retouch of the distal end) into hide scrapers, drills, or burins. This pattern is evidenced on Clovis points from Williamson, Flamingo Bay, Birckhead, and Gault^41–44^.

Redstone-74 (Fig. 15) exhibited a reddish microtrace (HT-5) that appears to be from a hafting arrangement using dry hide that was impregnated with ochre (see Kimball^61^: Fig. A68). This ochred dry hide trace (Hafting Trace-type 9) was first documented on an Early Archaic bifacial knife from the Main site in Kentucky (Kimball^45^:Appendix F-6; Fig. 9:C) (See Supplementary Fig. 2). It is also present on Clovis-27 (HT-1). Ochre/hematite is also documented on five Middle Paleoamerican bifaces at the Hipwater site in Michigan (Lovis et al*.*^46^:323) by Donahue^47^. Both lateral edges of this point were also used in heavy butchery, identified by the presence of fresh hide and meat polish along the edge with spots of bone polish. Light butchery is recognized by the presence of microtraces that resulted from cutting through fresh hide and meat.

**Figure 12:**
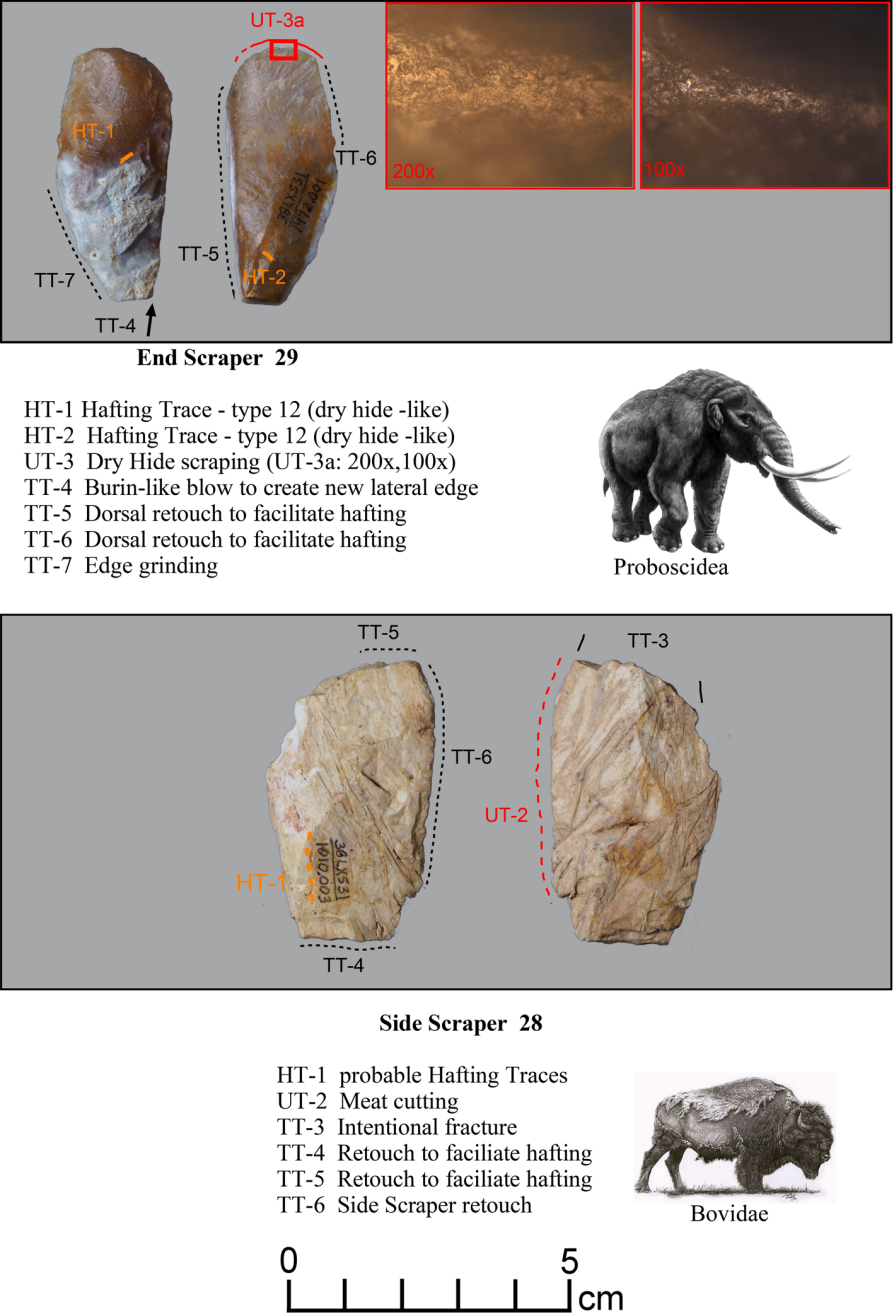
Microwear analysis for artifacts #29 and #28 (Table 2) showing the location and photomicrographs for hafting traces, dry hide scraping, meat cutting, edge grinding, retouching, and positive a CIEP result for each tool (Tables 1 and 2). The American Mastodon image is courtesy of La Brea Tar Pits, Los Angeles County Museum of Natural History and the Bovidae image (American Bison) by Chris Woolley is reproduced with permission of Connie Woolley.

Two scraping tools (Fig. 12) were found to possess preserved blood proteins for Proboscidea (Clovis End Scraper-29) and Bovidae (Clovis Side Scraper-28). After undergoing modifications to the proximal and both lateral edges, the end scraper was used hafted to scrape dry hide. The side scraper was modified at the proximal and distal ends to facilitate hafting and retouched along one lateral edge, which was then used to slice meat.

## Conclusions

These significant results carry implications for megafaunal exploitation and Paleoamerican archaeology of the region. First, to our knowledge, this is the only published immunological study for the eastern United States with evidence of Proboscidea and Equidae blood protein residues on Paleoamerican stone tool artifacts. Proboscidea blood residue is present on four Clovis artifacts, including an end scraper from a buried Clovis context, a large exotic chert Clovis knife, a Coastal Plain Chert Clovis, a metavolcanic Clovis point, and an early Paleoamerican Coastal Plain Chert Haw River point (Figs. 2 and 8; Table 1). Microwear evidence from a sample of tools that tested positive for Proboscidea, Equidae, and Bovidae, demonstrates that the tools were used in expected tasks related to projectile usage, as well as for butchery and fresh- and dry-hide scraping (Table 2).

Second, an earlier study for the region (Moore et al*.*^14^) did not find evidence of extinct megafauna on a small sample of Paleoamerican artifacts from South Carolina and Georgia and instead found evidence for only extant animals (e.g., Cervidae, Canidae, Leporidae, Felidae, and Ursidae), as well as Bovidae. The present inquiry is partially consistent with the earlier study by showing a strong presence of Bovidae blood residues (n = 9) on Paleoamerican stone tools including four Clovis, one Clovis-associated Side Scraper, three Redstone, and one Haw River (Figs. 4 and 8; Table 1). This suggests a heavy Paleoamerican focus on hunting bison and has implications for ecological conditions that likely existed in the Carolinas and Georgia at that time (e.g. the presence of open grasslands, potentially large migratory herds, and development and use of extensive game trails by Paleoamerican hunter-gatherers [e.g., Brooks et al*.*^48^; Moore and Irwin^49^]). There is a well-documented change in projectile point morphology between Clovis and Redstone points from blade-tip shapes effective for piercing and cutting (Clovis points) and piercing and penetrating (Redstone points) (Goodyear^50,51^) (Supplementary Fig. 3). The Redstone points would be more effective for repeatedly stabbing and withdrawing a spear as in dispatching animals in herds. Future Paleoamerican settlement and subsistence modeling in the Southeast should incorporate the findings of extensive hunting of bison and their implications.

Third, Proboscidea residue is present on an early Paleoamerican Haw River point (Fig. 2d). This point type is undated but the morphology and extensive weathered nature of the chert suggests an early Paleoamerican affiliation, possibly pre-Clovis (Painter^52,53^; Charles and Moore^54^; Whatley and Arena^55^; Gingerich and Childress^56^). The presence of Proboscidea suggests that Haw River points may be either coeval with or earlier than Clovis. Only more research including chronometric dating and documentation of this point type in an unambiguous stratigraphic context will resolve the question of where Haw River points fit chronologically.

Fourth, although the number of Middle Paleoindian points tested is too small (n = 16) for any definitive statement in this study, the lack of Proboscidea residue on post-Clovis Redstone, Cumberland, Quad, and Beaver Lake is tantalizing concerning the possible extirpation of mammoth and mastodon from the region at the onset of or during the early Younger Dryas. Equidae residue is present on Clovis (n = 2), Redstone (n = 1), and Beaver Lake (n = 1) points, suggesting that the Pleistocene horse persisted for a time after the onset of the Younger Dryas, consistent with likely post-Clovis-age horse bones associated with submerged Suwannee sites in northern Florida rivers (Dunbar and Vojnovksi^57^). See Supplementary Information “Background on Non-Clovis Paleoamerican Points”.

This study has demonstrated that immunological blood residue analysis of Paleoamerican stone tools can be used to address questions of human/animal interactions and extinctions even in areas of the United States where bone preservation is virtually nonexistent. The results of this study are also relevant to the ongoing debate about the potential effect of Paleoamerican hunting on megafaunal extinctions, which is unresolved and needs further study. To that end, the next step is to test a much larger sample of immediately post-Clovis types to further evaluate the timing of megafaunal extinctions in the Southeast and determine the relationship, if any, between extinction/extirpation of species, technological shifts in the archaeological record, and climatological changes associated with the onset of the Younger Dryas.

## Methods

(AINW) Residue Analysis Laboratory uses the technique of crossover immunoelectrophoresis (CIEP) to analyze protein residues extracted from the surface of stone artifacts and other objects. This technique has been widely used in forensic laboratories to determine the origin of bloodstains as evidence in criminal investigations and has fairly recently been adapted for use in archaeology to detect protein residues on stone tools. The CIEP method used by the AINW Residue Analysis Laboratory is based on techniques developed by the Royal Canadian Mounted Police Serology Laboratory in Toronto, Ontario (Culliford^58^; Newman^11^; Williams^59^).

The CIEP technique uses the immune (antibody‐antigen) reaction, the principle that all animals produce immunoglobulin proteins (antibodies) that recognize and bind with foreign proteins (antigens) as part of the body’s defense system. The ability of antibodies to precipitate antigens out of solution is the basis of CIEP analysis (Newman^11^:56). CIEP indicates the presence or absence of a particular antigen, and is not designed as a quantitative test. While other types of immunoassay have been used effectively to analyze blood protein residues under various conditions, the CIEP test is particularly suitable in that it is sensitive enough to detect proteins in concentrations of about two parts per million, does not require expensive or bulky equipment, is relatively fast (about 48 h per test), and can easily and efficiently accommodate multiple samples (Newman^11^:52).

Standard analytical procedures began with the extraction of proteins from the 120 artifacts. The artifact extracts were then placed singly into gels and tested against the nine chosen antisera with the CIEP technique. In addition to the artifact extracts, positive and negative control sera were run with each gel. This was done to determine if there were any contaminants or extraneous proteins that may give false positive results. If an anomalous result such as an extract reacting with a negative control serum is obtained, the extract solution is mixed with an equal volume of a 1% solution of a non-ionic detergent to increase chemical bonding specificity and is run through the CIEP process again. If a reaction still occurs after the addition of the non-ionic detergent, any reactions of those specimens to the antiserum are discounted. None of the extracts analyzed for this project reacted with the negative control.

The artifact extracts were tested against nine antisera including elephant, camel, horse, bovid, bear, deer, dog, cat, and rabbit (see Supplementary Table 1). The solutions are placed on a gel substrate and exposed to an electric current which causes the proteins to flow together. An immune reaction between the extract and the antiserum causes a precipitate to form, which is visible after being stained. See Supplementary Information “Blood Residue Analysis”.

### Microwear

Microwear analysis has demonstrated utility in the identification of activities undertaken at archaeological sites of cultures that used stone tools (Longo and Skakun^60^; van Gijn^40^). Although not discussed in detail here (see Kimball^61^), the method used in this study can identify the kind of material worked, the actions employed, and whether hafting was involved in the so-called “high power” microscopic approach, developed by Semenov^62^, clarified by Keeley^63^, and refined by others (see reviews by Juel Jensen^64^; Yerkes and Kardulias^65^; van Gijn^40,66^).

**Figure 13:**
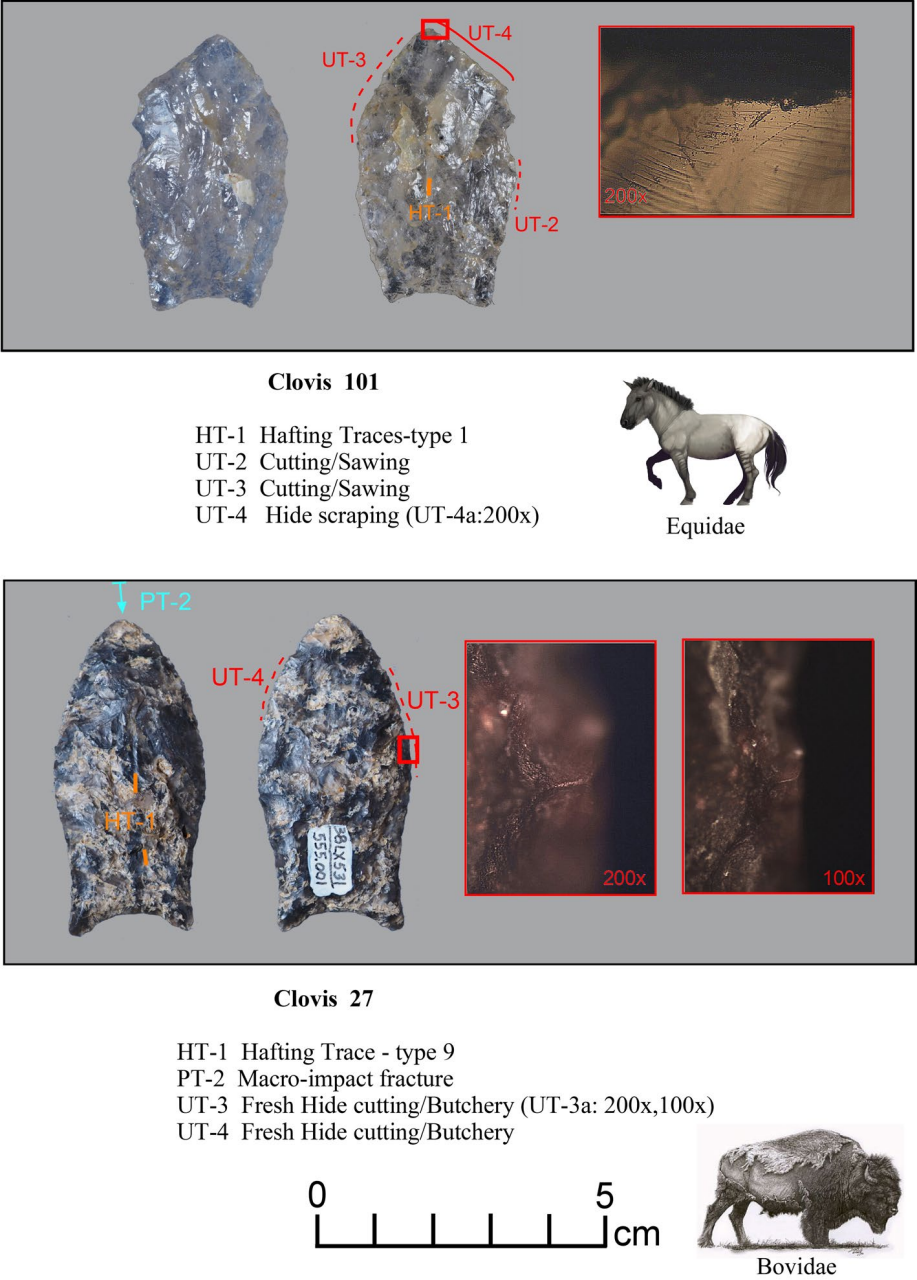
Microwear analysis for artifacts #101 and #27 (Table 2) showing locations and photomicrographs for hafting traces, cutting/sawing, hide scraping, micro-impact fracture, and fresh hide cutting/butchery, with positive CIEP results for each tool (Tables 1 and 2). The Equidae (Pleistocene horse) image is used with permission of Benji Paysnoe and is owned by the National Parks Service (NPS) and is in the public domain. The Bovidae image (American Bison) by Chris Woolley is reproduced with permission of Connie Wooley.

**Figure 14:**
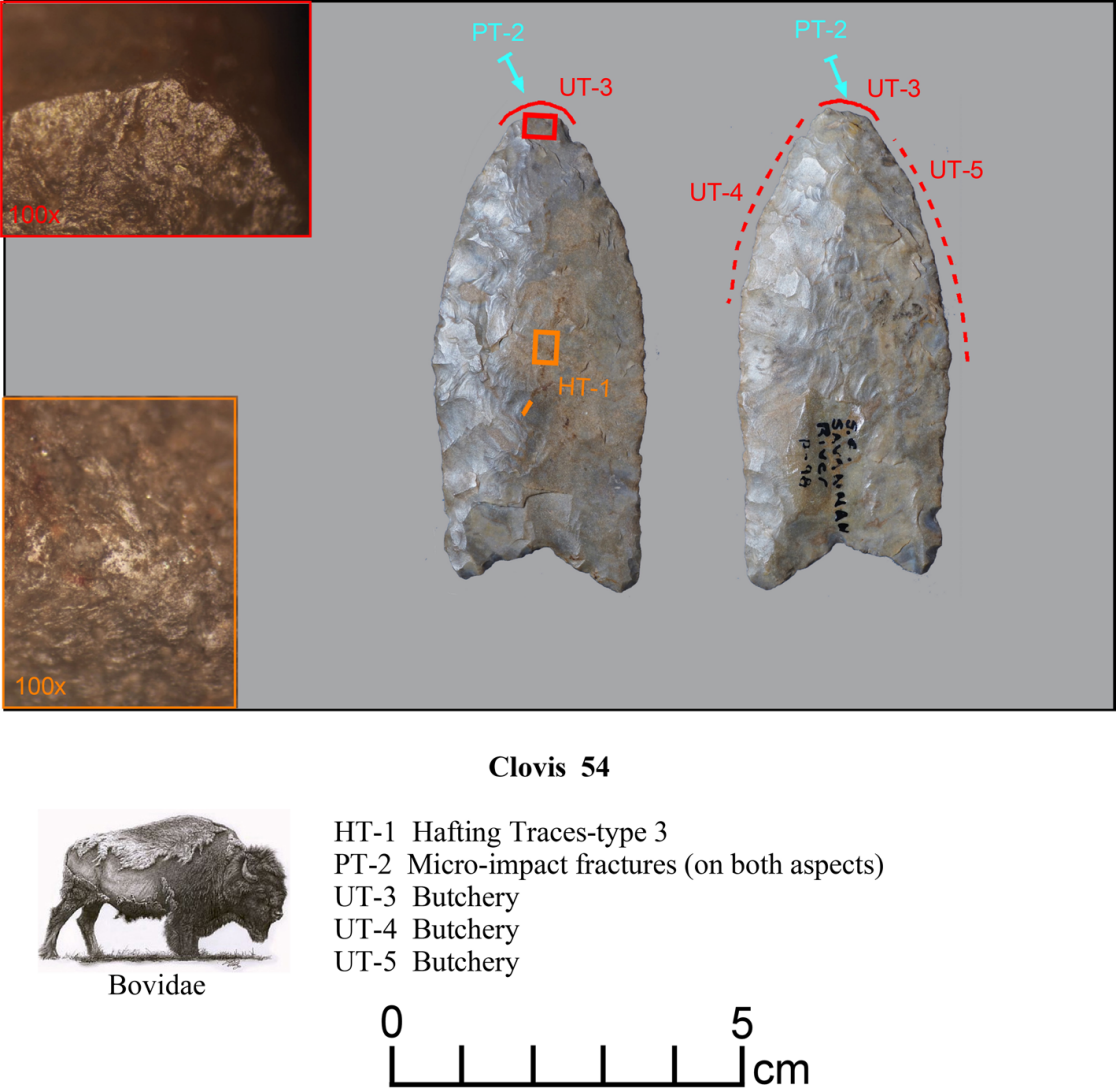
Microwear analysis for artifact #54 (Table 2) showing location and photomicrographs for hafting traces, butchery, and micro-impact fractures, with a positive CIEP result (Tables 1 and 2). The Bovidae image (American Bison) by Chris Woolley is reproduced with permission of Connie Woolley.

**Figure 15:**
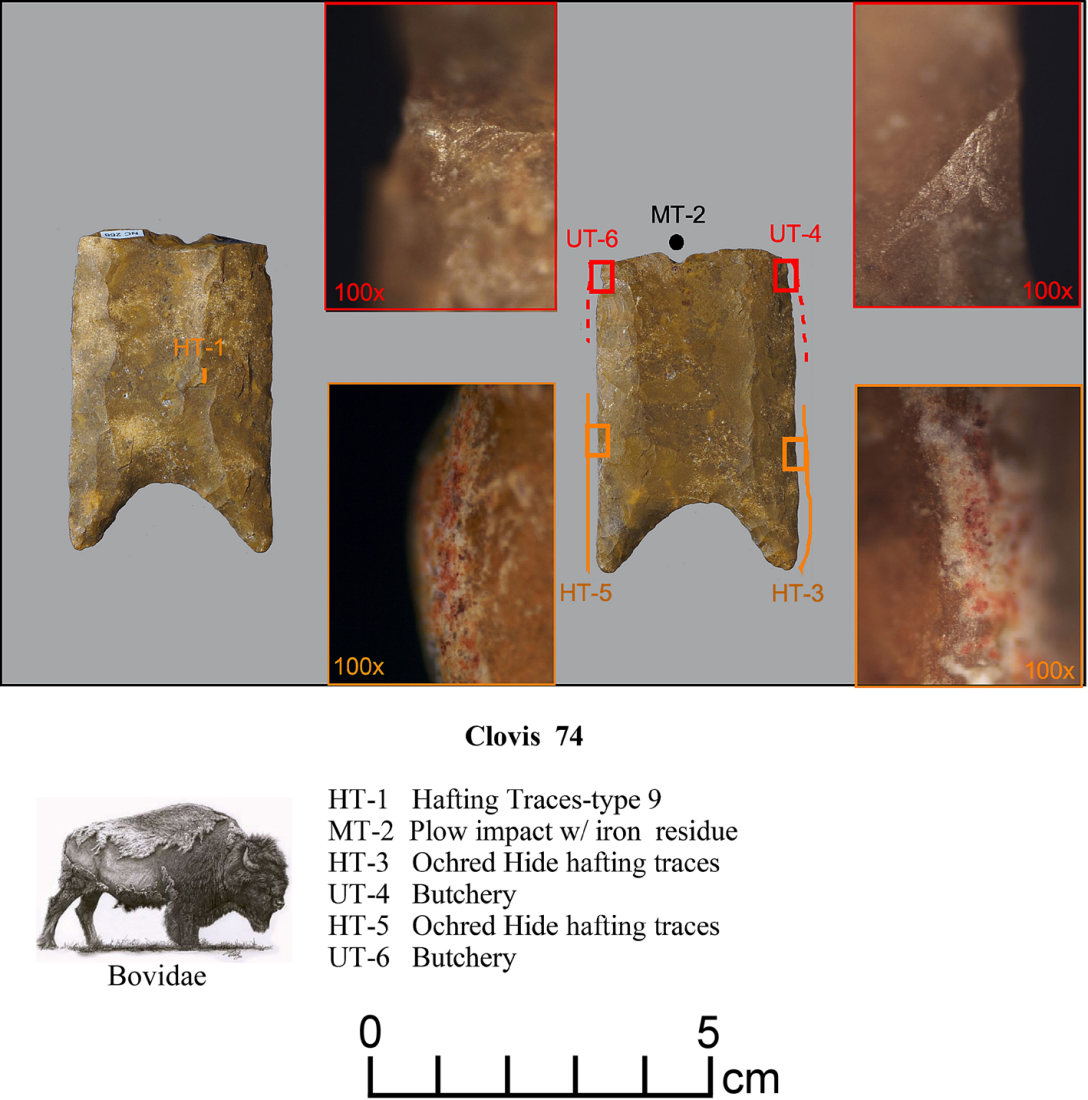
Microwear analysis for artifact #74 (Table 2) showing location and photomicrographs of hafting traces, butchery traces, and ochre residue, with a positive CIEP result (Tables 1 and 2). The Bovidae image (American Bison) by Chris Woolley is reproduced with permission of Connie Woolley.

These analyses were conducted using an Olympus BH metallurgical binocular microscope at 50, 100, and 200 power magnifications with incident light and a Dino-Lite AM4113TL-M40 USB microscope (5 × – 40 × magnification). Each archaeological specimen was digitally photographed and then ultrasonically cleaned in a commercial multi-purpose cleaner (Mr. Clean^®^) before microscopic examination. Generally, an archaeological specimen was scanned at 100 × to determine the existence of microtraces, then the areas with potential microtraces were then inspected at 200 ×. Once traces that are interpreted to be the consequence of manufacture, use, hafting, or post-discard alteration were identified, the spots (for discontinuous or spotty traces) or the distribution of these locations were drawn onto the artifact drawing. Codes employed in Figs. 10, 11, 12, 13, 14 and 15 to record the general class (use, manufacture, hafting, and post-discard traces) and the number of the microtraces for each tool, as follows: UT = use-trace; HT = hafting trace; PT = impact fracture due to projection; TT = technological-related to manufacture; ST = microwear trace due to sheath wear; and MT = microtrace not attributed to usage, hafting, projection or manufacture.

The identification of microtraces was made by reference to a collection of over 300 experiments conducted by Larry Kimball. The majority of these experiments and the observed microtraces due to use, projection, hafting, accidental breakage, trampling, and chemical alteration are documented in detail by the author elsewhere (Kimball^37,45,61,67–70^; Moore et al*.*^14^). Three of the eleven types of hafting traces described in detail by Kimball^61^:93–95; Kimball^61^: F4–F6) are observed in this analysis.

## Supplementary Information

Supplementary Information.

## Data Availability

All data needed for the evaluation of this paper are present in the paper and/or Supplementary Information. The artifacts studied in this paper have been returned to their respective owners and are not available from the authors for inspection. Additional data related to this paper may be requested from the corresponding author.
