# Supplementary material for: Paleoamerican exploitation of extinct megafauna revealed through immunological blood residue and microwear analysis, North and South Carolina, USA
Source: Sci Rep. 2023 Jun 10;13:9464. doi: 10.1038/s41598-023-36617-z (PMC10257692; doi:10.1038/s41598-023-36617-z)
Supplement: Supplementary file 1 — Supplementary Information. [file 41598_2023_36617_MOESM1_ESM.pdf]

Supplementary Information: Paleoamerican Exploitation of Extinct Megafauna Revealed through Immunological Blood Residue and Microwear Analysis, North and South Carolina, USA

Christopher R. Moore,[1,2]* Larry R. Kimball,[3] Albert C. Goodyear,[2] Mark J. Brooks,[2] I. Randolph Daniel, Jr.,[4] Allen West,[5] Sean G.Taylor,[1] Kiersten J. Weber,[1] John L. Fagan,[6] and Cam M. Walker[7]

[1]South Carolina Department of Natural Resources (SCDNR) Heritage Trust Program, Land, Water and Conservation Division, South Carolina Department of Natural Resources, PO Box 167, Columbia, SC 29202, USA, 919-218-0755. *MOORECR@mailbox.sc.edu; 803-238-1354. taylors@dnr.sc.gov; 803-261-4582. weberk@dnr.sc.gov. [2]South Carolina Institute for Archaeology and Anthropology, University of South Carolina 1321 Pendleton Street Columbia, SC 29208, USA, 919-218-0755. *MOORECR@mailbox.sc.edu; 803-237-7601; goodyear@mailbox.sc.edu. 803-422-8251. mjbrooks@mailbox.sc.edu. [3]Department of Anthropology, Appalachian State University, Boone, NC 28608, USA, 828-406-0218. kimballlr@appstate.edu. [4]Department of Anthropology, East Carolina University, Greenville, NC 27858, USA, 252-328-9455. danieli@ecu.edu. [5]Comet Research Group, Prescott, AZ USA, 928-632-7738. allen7633@aol.com. [6]Archaeological Investigations Northwest, 3510 N.E. 122nd Ave., Portland, OR 97230, USA, john@ainw.com. 503-761-6605. [7]WWAMI Medical Education Program, University of Wyoming, Dept. 4238 Health Sciences Bldg., Rm. 445E, 1000 E. University Avenue Laramie, WY 82071, USA, 307-766-6751. Cam.Walker@uwyo.edu.

*To whom correspondence should be addressed. E-mail: MOORECR@mailbox.sc.edu

Blood Residue Analysis

Blood Protein Residues. Blood is composed of red and white blood cells and serum, which is composed of about 150 different proteins including albumin, alpha, and beta globulins. Immunoglobulins are large, Y-shaped proteins with antigen-binding sites located on the V portion of the Y. There are several immunoglobulin molecules of different weights, sizes, and functions. The most common type (and the most pertinent for CIEP) is immunoglobulin G (IgG). Other less common varieties are immunoglobulin A (IgA), immunoglobulin D (IgD), immunoglobulin E (IgE), and immunoglobulin M (IgM). Some of these proteins can survive in the environment in a nonfunctional but immunologically identifiable form for long periods by forming a "covalently cross-linked proteinaceous mass with a high molecular weight" (Marlar *et al.*[1]:30). This combination of protein, fatty tissues, and soil particles is resistant to microbes and is markedly insoluble in water. It seems probable that the porosity and surface roughness of the artifact also aids in the preservation of protein residues. Experiments by AINW and others have identified blood residues from mammoth, bison, musk ox, horse, caribou, bear, duck, and trout on Paleoindian artifacts that may be as old as 11,500 years (Forgeng[2]; Loy and Dixon[3]; Williams[4]). Other studies suggest that protein residues can survive in recognizable form for as long as 40,000 years (Prager *et al.*[5]).

Artifacts can be examined under a binocular microscope (at around 240x maximum magnification) to identify probable residues, as well as cells, hair, and other tissues. Microscopic examination is not always effective as a screening technique as CIEP can still detect otherwise invisible residues. A common medical test for occult blood is sometimes effective when used to screen the extracted residue solution. However, the CEIP technique can detect residues in more dilute concentrations than is possible with the commonly available occult blood test.

The Immune Reaction. Immunological forensic tests owe their effectiveness to the antigen-antibody reaction, which allows very specific recognition and identification. Essentially, any molecule that can bind to an antibody is an antigen. For archaeological purposes, the antigen is an unknown protein adhering to an artifact after its use. Antigens are foreign proteins that, when introduced into the bloodstream of an animal, stimulate the immune system of the animal to produce antibodies (most commonly IgG protein molecules) with specific binding sites that match corresponding sites on the foreign antigen. Polyclonal antibodies, which bind to multiple

sites on the antigen and therefore have a high rate of successful matching to unknown proteins, are the most commonly used reactants in CIEP. The meeting of antigen and antibody forms a very strong bond between the two proteins. The visible line formed in a positive CIEP reaction occurs when an antigen with multiple binding sites matches a group of polyclonal antibodies, binds with them, and causes the proteins to precipitate out of solution (Marlar *et al.*[1]:28).

Antisera. The antisera used in AINW's CIEP analysis are obtained from commercial laboratories (Supplementary Table 1). A forensic antiserum is made by injecting a host animal, typically a goat or rabbit, with a protein solution obtained from another animal. The immune system of the host animal produces antibodies (mainly IgG) in reaction to the foreign antigen. Blood serum drawn from the host animal is purified and tested to determine the range of reactivity of the antiserum. The purified antiserum is then freeze-dried for storage and shipment. After receipt of a new lot of antiserum, the AINW laboratory routinely tests each antiserum against representative specimens from up to 32 different animal species.

Ancient protein residues are often difficult to extract from the artifacts that have preserved them. The AINW Residue Analysis Laboratory uses a 5% ammonia solution, which has been used for similar applications in forensic medicine (Dorrill and Whitehead[6]; Kind and Cleevely[7]). Ammonia is generally more effective in lifting old and partially denatured blood proteins than other solvents (Newman[8]). A small amount of the ammonia solution is applied to the artifact in a plastic tray, and the tray and artifact are placed in an ultrasonic bath (Branson 2200) for 30 minutes or longer. The artifact in solution is then placed on a mechanical rotator (Thermolyne Rotomix) for an additional ten minutes.

Artifacts too large for ultrasonic extraction may be placed on the rotator for 30 minutes or longer. Residues from soil samples can also be extracted using variations of these methods. The extraction solution is then drawn off and stored in an airtight microcentrifuge tube. The extracts are centrifuged to clarify the sample and refrigerated; the CIEP test is run as soon as possible after extraction. The extracts may be frozen immediately if testing is to be delayed for more than one week.

AINW's CIEP method uses an agarose gel as a substrate. Standard analysis procedures begin with extracting residues from the artifacts with a 5% ammonia solution. The artifact

extracts are then placed singly into gels and tested against the antisera selected for these tests with the CIEP technique. In addition to the artifact extracts, positive and negative control sera are run with each gel. This is done to determine if there are any contaminants or extraneous proteins that may give false positive results. If an anomalous result such as an extract reacting with multiple antisera or to a negative control serum is obtained, the extract solution is mixed with an equal volume of a 1% solution of a non-ionic detergent to increase chemical bonding specificity and is run through the CIEP process again. If a reaction still occurs after the addition of the non-ionic detergent, any reactions of those specimens to the antisera are discounted. Experiments at AINW have implicated plant pitch used in hafting prehistoric stone tools as a possible cause of some cross or non-specific reactions. Electrophoresis is used to drive the antigens and antibodies together. The gel substrates are placed in acrylic electrophoresis tanks filled with barbital buffer solution, then attached to the regulated H.V. power source. The antibodies move toward the cathode because of the overall negative charge on the molecule, while the antigens move toward the anode. A precipitate is formed where the proteins meet and bond in the area between the wells, visible as a white line or arc (Culliford[9]). The gel is soaked overnight in saline to stabilize the reaction, then dried and stained with a standard protein stain as a permanent record of the CIEP results. The dried and stained gel is then backlit on a light table, and examined under magnification for the presence of precipitate lines, indicating positive reactions. After testing, the extracts are frozen and stored for one year in case additional testing is requested.

Background on Non-Clovis Paleoamerican Points

Haw River. Unidentified small lanceolates (USLs) have been noted in many areas of the Southeast for a number of years as representing something different than Clovis but likely of early Paleoamerican age (Supplementary Figure 4). In South Carolina, what we now refer to as Haw River points were initially called “Heart Points” by Tommy Charles during years of documenting private collections across the state (Charles and Moore[10]). In North Carolina, a morphologically similar point called the Hardaway Blade type was recognized by Coe[11] at the base of the Hardaway Site. Following Goodyear[12], Daniel[13] suggests some Hardaway Blades are likely Hardaway Dalton preforms but other such bifaces appear to be finished points and may be a separate unknown point type that could predate both Hardaway Dalton and Clovis. In South Carolina, Haw River points made of Coastal Plain Chert (CPC) are excessively weathered, even

when compared to Clovis points of the same material, and provide some support for a very early Paleoamerican affiliation for this point type. Both Meadowcroft and Cactus Hill have similar small lanceolate points with claims for pre-Clovis affiliation (Adovasio[14]; McAvoy and McAvoy[15]). At the present time "Haw Rivers" have only a morphological point status but they are widely recognized along the East Coast and are presented as a possible type in several publications for sites in Virginia, North Carolina, South Carolina, and Georgia (Painter[16,17]; Gingerich and Childress[18]; Charles and Moore[10]; Whatley and Arena[19]). Thus, they are recognizable by some archaeologists in multiple states and are technologically distinct from Clovis. Associated chronometric dates for this point type do not yet exist and excavations with Haw River points in a clear stratigraphic context are needed to resolve the chronostratigraphic position of this point type relative to other Paleoamerican types.

Redstone. Redstone points have characteristic deeply concave bases, triangular blade margins, and long flutes that can extend, in some cases, to near the distal end of the point. Goodyear[20] has argued that fluting these points is instrument-assisted. Redstone points in North and South Carolina are similar to those from elsewhere in the mid-South (Cambron and Hulse[21]). A post-Clovis affiliation for Redstone points has been assumed based on point morphology that closely resembles both Folsom and Gainey points. In her chapter in the Smallwood and Jennings[22] edited volume, Julie Morrow declares that Redstones are in reality Gainey points (Morrow[23]). "Although first described from examples in Alabama (Cambron and Hulse[21]), Redstone Points have a broad distribution across the Midsouth. Other than Tennessee (Broster and Norton[24]), they occur in South Carolina, Virginia, and North Carolina and are equated with other fully fluted points like Folsom, Gainey, and Cumberland (Goodyear[25]). Redstones are Gainey points that occur in the Southeast" (Morrow[23]). Goodyear[25] speculates that the elongated trianguloid blade could be an adaptation to hunting elk and bison after the extinction of Proboscideans. Until very recently, Redstone points lacked any associated chronometric dates. However, at Cactus Hill, wood charcoal was dated from associated hearths with *in-situ* deeply concave base (DCB) fluted points (i.e., Redstone) and provided ^{14}C dates that range from ca. 10.9 to 10.8 BP (McAvoy and McAvoy[26]: Table 5.8). This puts Redstone at the tail end of Clovis with a range that likely extends for a yet unknown

amount of time earlier and later. More dating is needed to constrain the timing and usage of this type.

Cumberland. Like Redstone points, Cumberland points are full-facial fluted with one or multiple flutes on each face, are basally ground, and often are slightly waisted with distinct ears and a shallower basal concavity than Redstone (Daniel[13]; Cambron and Hulse[21]). Based on morphology, Cumberland points are presumed to fall into the Middle Paleoindian time frame with other similar full-fluted and waisted points (Tune[27]). As of this writing, no associated radiocarbon dates exist for Cumberland.

Simpson. Simpson points are common in Florida and the Georgia Coastal Plain, but a small number can be found in the Carolinas. This type was described by Bullen[28] as: "A wide-bladed, relatively narrow-waisted, fairly thin, concave based, medium to large sized point with grinding on the bottom and waisted edges. Basal ears are present but are not as developed as in the Suwannee point. Basal thinning is present but, also, is not well developed. Workmanship is good to fair." Simpson points differ from Suwannee points because of their extremely waisted appearance and lack of well-developed ears." Meeks and Anderson[29] place Simpson points in the Middle Paleoindian time frame between 12,600 and 12,000 cal. BP; however, as of the writing of this paper, no chronometric dates exist for this point type and Dunbar[30] considers at least some Simpson variants to potentially pre-date Clovis in Florida.

Beaver Lake. As described by Cambron and Hulse[21] and DeJarnette *et al.*[31], this is a medium-sized, auriculated point with recurvate blade edges. DeJarnette *et al.*[31] wrote, "the cross-section is usually biconvex, but one or both faces may be median ridged. The blade is recurvate-constricted in the hafting area and above the auricles. The distal end is usually acute. The auriculated hafting area is expanded-rounded. The basal edge is usually thinned and incurvate but may be straight. The hafting constriction and basal edge are usually ground." The shallow random flaking is usually employed to shape the faces and sometimes produces a median ridge. Secondary retouch flake scars are usually long, evenly spaced, and struck off on alternate faces, resulting in an irregular pattern along the blade edges. This retouch appears to have been accomplished with indirect percussion or pressure flaking."

DeJarnette *et al.*[31] wrote, “the type was named for the Beaver Lake area in Limestone County, Alabama. The type has been called Unfluted Cumberland in several papers, especially Soday and Cambron[32]. All evidence indicates the type dates from 10,000 B.P. to an unknown earlier time. It is considered to be a transitional Paleo-Indian type”. DeJarnette, Kurjack, Cambron, and Hulse named the point in 1962 for examples that were recovered from the Stanfield-Whorley Bluff Shelter, Colbert County, Alabama. Like other waisted lanceolates, this type is assumed to fit within the Middle Paleoindian time frame but as with other Paleoamerican points found in the Southeast, chronometric dates for this type do not yet exist.

Supplementary Tables

Supplementary Table 1. Antiserum chart for Archaeological Investigations Northwest, Inc.

COMPANY	ANTISERUM	HOST	REACTS WITH
BLI*	BIRD	goat	Class Aves: dove, duck, sparrow, chicken, ostrich, emu, goose, macaw
	BOTTLENOSE DOLPHIN	rabbit	Family Delphinidae: dolphins, less strongly with porpoises and toothed whales
	FERRET	rabbit	Family Mustelidae: ferret, otter, badger, mink, stoat, wolverine, marten
	SHEEP	rabbit	Genus Ovis: domestic sheep, bighorn sheep
	WHITE WHALE	rabbit	Family Monodontidae: belugas and narwhals, porpoises
BYT*	DUCK	rabbit	Family Anatidae: swans, geese and ducks
CBI*	TROUT	rabbit	Subfamily Salmoninae: salmon, steelhead, rainbow trout, char
MP*	BOVINE	rabbit	Family Bovidae: domestic cow, bison
	CAT	goat	Family Felidae: cat, mountain lion, lynx, bobcat
	CHICKEN	rabbit	Order Galliformes, Order Anseriformes, Order Columbiformes
	DEER	goat	Family Cervidae: white-tail and mule deer, elk, moose, caribou
	DOG	rabbit	Family Canidae: domestic dog, coyote, wolf, fox
	GOAT	rabbit	Bovid Subfamilies Bovinae and Caprinae, less strongly with cervids
	GUINEA PIG	goat	Order Rodentia: guinea pig, porcupine, beaver
	HORSE	goat	Family Equidae: horse, donkey, mule, extinct equids
	HUMAN	goat	Order Primates: humans, apes, monkeys
	MOUSE	goat	Order Rodentia: mice, rats
	RABBIT	goat	Family Leporidae: rabbit, jackrabbit
	RAT	goat	Order Rodentia: rats, mice, squirrels
	SWINE (PIG)	goat	Family Suidae: pig, hog, warthog
NIL*	PIGEON	rabbit	Order Columbiformes: pigeons, doves
Triple J Farms (custom)	BEAR	goat	Family Ursidae: black bear, brown bear, grizzly
	CAMEL	goat	Order Artiodactyla: camelids, bovids, cervids, antilocaprids
	HIPPO	goat	Family Hippopotamidae: extant and excinct hippo species
	OSTRICH	goat	Family Struthionidae: extant and extinct ostrich species
	RHINO	goat	Family Rhinocerotidae: white, black, Indian, Javan, Sumatran, and extinct rhinoceros
	ELEPHANT	goat	Asian and African elephants and extant and extinct members of Order Proboscidea

*Notes: BLI = Bethyl Laboratories, Inc., BYT = Biorbyt Laboratories, Inc., CBI = Cocalico Biologicals, Inc., IR = Innovative Research, Inc., MP = MP Biomedicals, LLC, NIL = Nordic Immunological Laboratories

Supplementary Table 2. Information on artifact context and ownership.

Analysis ID	Artifact ID	Type	Context	Owner/Curator	Type
**1**	JC 8	Clovis	Surface	Hampton County Museum	Musuem
**2**	JC 7	Clovis	Surface	Hampton County Museum	Musuem
**3**	JC 418	Simpson	Surface	Hampton County Museum	Musuem
**4**	JC 415	Clovis	Surface	Hampton County Museum	Musuem
**5**	JC 425	Clovis	Surface	Hampton County Museum	Musuem
**6**	JC 9	Clovis	Surface	Hampton County Museum	Musuem
**7**	JC 423	Clovis	Surface	Hampton County Museum	Musuem
**8**	JC 420	Haw River	Surface	Hampton County Museum	Musuem
**9**	JC 6	Redstone	Surface	Hampton County Museum	Musuem
**10**	JC 10	Clovis	Surface	Hampton County Museum	Musuem
**11**	—	Clovis	Surface	Hampton County Museum	Musuem
**12**	JC 417	Clovis	Surface	Hampton County Museum	Musuem
**13**	JC 419	Haw River	Surface	Hampton County Museum	Musuem
**14**	JC 422	Haw River	Surface	Hampton County Museum	Musuem
**15**	JC 408	Haw River	Surface	Hampton County Museum	Musuem
**16**	JC 407	Haw River	Surface	Hampton County Museum	Musuem
**17**	JC 411	Haw River	Surface	Hampton County Museum	Musuem
**18**	JC 406	Haw River	Surface	Hampton County Museum	Musuem
**19**	JC 400	Haw River	Surface	Hampton County Museum	Musuem
**20**	JC 404	Haw River	Surface	Hampton County Museum	Musuem
**21**	JC 410	Haw River	Surface	Hampton County Museum	Musuem
**22**	JC 402	Haw River	Surface	Hampton County Museum	Musuem
**23**	JC 413	Haw River	Surface	Hampton County Museum	Musuem
**24**	JC 401	Haw River	Surface	Hampton County Museum	Musuem
**25**	JC 403	Haw River	Surface	Hampton County Museum	Musuem
**26**	JC 405	Haw River	Surface	Hampton County Museum	Musuem
**27**	38LX531a	Clovis	Excavated	Dominion Energy	Company
**28**	38LX531b	Clovis Side Scraper	Excavated	Dominion Energy	Company
**29**	38LX531c	Clovis End Scraper	Excavated	Dominion Energy	Company
**30**	SC680	Clovis	Surface	SCIAA/SEPAS	University of South Carolina
**31**	SC682	Clovis	Surface	SCIAA/SEPAS	University of South Carolina
**32**	SC679	Clovis	Surface	SCIAA/SEPAS	University of South Carolina
**33**	SC358	Clovis	Surface	SCIAA/SEPAS	University of South Carolina
**34**	SC681	Clovis	Surface	SCIAA/SEPAS	University of South Carolina
**35**	SC547	Clovis	Surface	SCIAA/SEPAS	University of South Carolina
**36**	SC670	Clovis	Surface	SCIAA/SEPAS	University of South Carolina
**37**	SC766	Clovis	Surface	SCIAA/SEPAS	University of South Carolina
**38**	SC215	Redstone	Surface	SCIAA/SEPAS	University of South Carolina
**39**	SC488	Clovis	Surface	SCIAA/SEPAS	University of South Carolina
**40**	LS 5141	Clovis	Surface	SCIAA/SEPAS	University of South Carolina
**41**	LS 5137	Haw River	Surface	SCIAA/SEPAS	University of South Carolina
**42**	LS 5136	Haw River	Surface	SCIAA/SEPAS	University of South Carolina
**43**	LS (2018)	Haw River	Surface	SCIAA/SEPAS	University of South Carolina
**44**	P-77	Clovis	Surface	SCIAA/SEPAS	University of South Carolina
**45**	SC445	Clovis	Surface	SCIAA/SEPAS	University of South Carolina
**46**	P-7	Redstone	Surface	SCIAA/SEPAS	University of South Carolina
**47**	P-76	Clovis	Surface	SCIAA/SEPAS	University of South Carolina
**48**	P-15	Clovis	Surface	SCIAA/SEPAS	University of South Carolina
**49**	P-96	Clovis	Surface	SCIAA/SEPAS	University of South Carolina
**50**	SC382	Clovis	Surface	SCIAA/SEPAS	University of South Carolina
**51**	P-94	Clovis	Surface	SCIAA/SEPAS	University of South Carolina
**52**	SC641	Clovis	Surface	SCIAA/SEPAS	University of South Carolina
**53**	SC442	Clovis	Surface	SCIAA/SEPAS	University of South Carolina
**54**	SC444	Clovis	Surface	SCIAA/SEPAS	University of South Carolina
**55**	SC 450 or SC451	Clovis	Surface	SCIAA/SEPAS	University of South Carolina
**56**	P-80	Clovis	Surface	SCIAA/SEPAS	University of South Carolina
**57**	SC422	Clovis	Surface	SCIAA/SEPAS	University of South Carolina
**58**	P-95	Clovis	Surface	SCIAA/SEPAS	University of South Carolina
**59**	SC440	Clovis	Surface	SCIAA/SEPAS	University of South Carolina
**60**	P-79	Clovis	Surface	SCIAA/SEPAS	University of South Carolina

Supplementary Table 2 (cont). Information on artifact context and ownership.

Analysis ID	Artifact ID	Type	Context	Owner/Curator	Type
**61**	S452	Clovis	Surface	SCIAA/SEPAS	University of South Carolina
**62**	P-81	Clovis	Surface	SCIAA/SEPAS	University of South Carolina
**63**	P-99	Clovis	Surface	SCIAA/SEPAS	University of South Carolina
**64**	AB#1	Clovis	Surface	Anthony Bowen	Private Collection
**65**	AB#2	Clovis	Surface	Anthony Bowen	Private Collection
**66**	AB#3	Clovis	Surface	Anthony Bowen	Private Collection
**67**	AB#4	Redstone	Surface	Anthony Bowen	Private Collection
**68**	AB#5	Haw River	Surface	Anthony Bowen	Private Collection
**69**	AB#6	Clovis	Surface	Anthony Bowen	Private Collection
**70**	SC359	Redstone	Surface	Fort Jackson	Military Base
**71**	SC741	Clovis	Surface	Fort Jackson	Military Base
**72**	SC287	Cumberland	Surface	SCIAA/SEPAS	University of South Carolina
**73**	SC782	Haw River	Surface	SCIAA/SEPAS	University of South Carolina
**74**	NC266	Redstone	Surface	SCIAA/SEPAS	University of South Carolina
**75**	Overstreet #9	Clovis	Surface	John Kolmar	Private Collection
**76**	SC-632	Clovis	Surface	Chris Young	Private Collection
**77**	RP-#103	Clovis	Surface	Laurens County Museum	Museum
**78**	SC388	Clovis	Surface	Laurens County Museum	Museum
**79**	38LX283	Redstone	Excavated	SCDNR	SC Department of Natural Resources
**80**	38RD18	Clovis	Surface	SCDNR	SC Department of Natural Resources
**81**	Hendrix1	Clovis	Surface	Dennis Hendrix	Private Collection
**82**	Hendrix2	Clovis	Surface	Dennis Hendrix	Private Collection
**83**	Hendrix3	Clovis	Surface	Dennis Hendrix	Private Collection
**84**	Hendrix4	Clovis	Surface	Dennis Hendrix	Private Collection
**85**	Arena1	Beaver Lake	Surface	John Arena	Private Collection
**86**	Arena2	Clovis	Surface	John Arena	Private Collection
**87**	Arena3	Quad	Surface	John Arena	Private Collection
**88**	Arena4	Clovis	Surface	John Arena	Private Collection
**89**	Arena5	Clovis	Surface	John Arena	Private Collection
**90**	Arena6	Redstone	Surface	John Arena	Private Collection
**91**	NC186	Clovis	Surface	Phelps Archaeology Laboratories, ECU	East Carolina University
**92**	NC187	Clovis	Surface	Phelps Archaeology Laboratories, ECU	East Carolina University
**93**	NC303	Redstone	Surface	Phelps Archaeology Laboratories, ECU	East Carolina University
**94**	VS1	Clovis	Surface	Steen Family	Private Collection
**95**	VS2	Clovis	Surface	Steen Family	Private Collection
**96**	VS3	Clovis	Surface	Steen Family	Private Collection
**97**	VS4	Clovis	Surface	Steen Family	Private Collection
**98**	VS5	Clovis	Surface	Steen Family	Private Collection
**99**	VS6	Clovis	Surface	Steen Family	Private Collection
**100**	VS7	Clovis	Surface	Steen Family	Private Collection
**101**	VS8	Clovis	Surface	Steen Family	Private Collection
**102**	VS9	Clovis	Surface	Steen Family	Private Collection
**103**	P-97	Redstone	Surface	SCIAA/SEPAS	University of South Carolina
**104**	SC750	Clovis	Surface	SCIAA/SEPAS	University of South Carolina
**105**	SC617	Waisted Lanceolate	Surface	SCIAA/SEPAS	University of South Carolina
**106**	JL1	Clovis	Surface	Jim Legg	Private Collection
**107**	JL2	Redstone	Surface	Jim Legg	Private Collection
**108**	JL3	Clovis	Surface	Jim Legg	Private Collection
**109**	LS 5197	Haw River	Surface	SCIAA/SEPAS	University of South Carolina
**110**	LS 5206	Haw River	Surface	SCIAA/SEPAS	University of South Carolina
**111**	LS 5213	Haw River	Surface	SCIAA/SEPAS	University of South Carolina
**112**	LS 5205	Haw River	Surface	SCIAA/SEPAS	University of South Carolina
**113**	LS 5215	Haw River	Surface	SCIAA/SEPAS	University of South Carolina
**114**	LS 5207	Haw River	Surface	SCIAA/SEPAS	University of South Carolina
**115**	LS 5210	Haw River	Surface	SCIAA/SEPAS	University of South Carolina
**116**	LS 5202	Haw River	Surface	SCIAA/SEPAS	University of South Carolina
**117**	LS 5199	Haw River	Surface	SCIAA/SEPAS	University of South Carolina
**118**	LS 5204	Haw River	Surface	SCIAA/SEPAS	University of South Carolina
**119**	LS5212	Haw River	Surface	SCIAA/SEPAS	University of South Carolina
**120**	BZ1	Clovis Knife	Surface	Ben Ziegler	Private Collection

Supplementary Table 3. Artifacts with Negative Immunological Results.

Analysis ID	Artifact ID	County/State	Type	Cultural Affiliation	[1]Raw Material	CIEP Results
**2**	JC 7	Hampton/SC	Clovis	Early Paleoindian	CPC	Negative
**3**	JC 418	Hampton/SC	Simpson	Middle Paleoindian	CPC	Negative
**5**	JC 425	Hampton/SC	Clovis	Early Paleoindian	CPC	Negative
**6**	JC 9	Hampton/SC	Clovis	Early Paleoindian	CPC	Negative
**7**	JC 423	Hampton/SC	Clovis	Early Paleoindian	CPC	Negative
**8**	JC 420	Hampton/SC	Haw River	Early Paleoindian	CPC	Negative
**9**	JC 6	Hampton/SC	Redstone	Middle Paleoindian	CPC	Negative
**10**	JC 10	Hampton/SC	Clovis	Early Paleoindian	CPC	Negative
**11**	JC	Hampton/SC	Clovis	Early Paleoindian	CPC	Negative
**12**	JC 417	Hampton/SC	Clovis	Early Paleoindian	CPC	Negative
**13**	JC 419	Hampton/SC	Haw River	Early Paleoindian	CPC	Negative
**14**	JC 422	Hampton/SC	Haw River	Early Paleoindian	CPC	Negative
**15**	JC 408	Hampton/SC	Haw River	Early Paleoindian	CPC	Negative
**16**	JC 407	Hampton/SC	Haw River	Early Paleoindian	CPC	Negative
**17**	JC 411	Hampton/SC	Haw River	Early Paleoindian	CPC	Negative
**18**	JC 406	Hampton/SC	Haw River	Early Paleoindian	CPC	Negative
**19**	JC 400	Hampton/SC	Haw River	Early Paleoindian	CPC	Negative
**20**	JC 404	Hampton/SC	Haw River	Early Paleoindian	CPC	Negative
**21**	JC 410	Hampton/SC	Haw River	Early Paleoindian	CPC	Negative
**22**	JC 402	Hampton/SC	Haw River	Early Paleoindian	CPC	Negative
**23**	JC 413	Hampton/SC	Haw River	Early Paleoindian	CPC	Negative
**24**	JC 401	Hampton/SC	Haw River	Early Paleoindian	CPC	Negative
**25**	JC 403	Hampton/SC	Haw River	Early Paleoindian	CPC	Negative
**26**	JC 405	Hampton/SC	Haw River	Early Paleoindian	CPC	Negative
**30**	SC680	Allendale/SC	Clovis	Early Paleoindian	CPC	Negative
**31**	SC682	Allendale/SC	Clovis	Early Paleoindian	CPC	Negative
**32**	SC679	Allendale/SC	Clovis	Early Paleoindian	CPC	Negative
**33**	SC358	Allendale/SC	Clovis	Early Paleoindian	CPC	Negative
**34**	SC681	Allendale/SC	Clovis	Early Paleoindian	CPC	Negative
**35**	SC547	Allendale/SC	Clovis	Early Paleoindian	MTV	Negative
**36**	SC670	Allendale/SC	Clovis	Early Paleoindian	CPC	Negative
**39**	SC488	Allendale/SC	Clovis	Early Paleoindian	CPC	Negative
**40**	LS 5141	Allendale/SC	Clovis	Early Paleoindian	CPC	Negative
**41**	LS 5137	Allendale/SC	Haw River	Early Paleoindian	CPC	Negative
**42**	LS 5136	Allendale/SC	Haw River	Early Paleoindian	CPC	Negative
**43**	LS (2018)	Allendale/SC	Haw River	Early Paleoindian	CPC	Negative
**44**	P-77	McCormick/SC	Clovis	Early Paleoindian	Ch	Negative
**47**	P-76/SC407	Edgefield,/SC	Clovis	Early Paleoindian	CPC	Negative
**48**	P-15	McCormick/SC	Clovis	Early Paleoindian	CPC	Negative
**49**	P-96/SC385	Edgefield/SC	Clovis	Early Paleoindian	CPC	Negative
**50**	SC382	Beaufort/SC	Clovis	Early Paleoindian	Jas	Negative
**51**	P-94/SC442	Lexington/SC	Clovis	Early Paleoindian	Ch	Negative
**53**	SC442	Lexington/SC	Clovis	Early Paleoindian	Ch	Negative
**55**	SC450 or SC451	Clarendon/SC	Clovis	Early Paleoindian	BVT	Negative

[1]Raw Materials types include Coastal Plain Chert (CPC), Chert (Ch), Metavolcanic (MTV), Rhyolite (Rhy), Flow-Banded Rhyolite (FBR), Aphyric Rhyolite (AR), Plagioclase-Porphyritic Rhyolite (PPR), Quartz (Q), Crystal Quartz, (CQ), Green Vitric Tuff (GVT), Black Vitric Tuff (BVT), Jasper (Jas), and Black Mingo Chert (BMC).

Supplementary Table 3 (cont). Artifacts with Negative Immunological Results.

Analysis ID	Artifact ID	County/State	Type	Cultural Affiliation	[1]Raw Material	CIEP Results
**56**	P-80	Sumter/SC	Clovis	Early Paleoindian	CPC	Negative
**57**	SC422	Edgefield/SC	Clovis	Early Paleoindian	MTV	Negative
**58**	P-95	Lake Marion/SC	Clovis	Early Paleoindian	CPC	Negative
**60**	P-79/SC384	Lake Marion/SC	Clovis	Early Paleoindian	CPC	Negative
**62**	P-81/SC409	Lake Marion	Clovis	Early Paleoindian	Ch	Negative
**63**	P-99/SC386	Clarendon/SC	Clovis	Early Paleoindian	CPC	Negative
**64**	AB#1	Darlington/SC	Clovis	Early Paleoindian	MTV	Negative
**65**	AB#2	Darlington/SC	Clovis	Early Paleoindian	GVT	Negative
**67**	AB#4	Darlington/SC	Redstone	Middle Paleoindian	BVT	Negative
**68**	AB#5	Darlington/SC	Haw River	Early Paleoindian	MTV	Negative
**69**	AB#6	Darlington/SC	Clovis	Early Paleoindian	Chert	Negative
**70**	SC359	Richmond/SC	Redstone	Early Paleoindian	AR	Negative
**71**	SC741	Richmond/SC	Clovis	Early Paleoindian	Rhy	Negative
**72**	SC287	Lexington/SC	Cumberland	Middle Paleoindian	Ch	Negative
**73**	SC782	Abbeville/SC	Haw River	Early Paleoindian	MTV	Negative
**75**	Overstreet #9	Edgefield/SC	Clovis	Early Paleoindian	CPC	Negative
**76**	SC632	Kershaw/SC	Clovis	Early Paleoindian	MTV	Negative
**77**	RP-#103	Surry/NC	Clovis	Early Paleoindian	Jas	Negative
**78**	SC388	Lexington/SC	Clovis	Early Paleoindian	CPC	Negative
**79**	38LX283	Lexington/SC	Redstone	Middle Paleoindian	Ch	Negative
**81**	Hendrix1	Jasper/SC	Clovis	Early Paleoindian	CPC	Negative
**82**	Hendrix2	Orangeburg/SC	Clovis	Early Paleoindian	CPC	Negative
**83**	Hendrix3	Orangeburg/SC	Clovis	Early Paleoindian	CPC	Negative
**84**	Hendrix4	Bamburg/SC	Clovis	Early Paleoindian	CPC	Negative
**86**	Arena2	Edgefield/SC	Clovis	Early Paleoindian	CPC	Negative
**87**	Arena3	Aiken/SC	Quad	Middle Paleoindian	Ch	Negative
**88**	Arena4	Saluda/SC	Clovis	Early Paleoindian	Jas	Negative
**89**	Arena5	Burke/GA	Clovis	Early Paleoindian	CPC	Negative
**90**	Arena6	Edgefield/SC	Redstone	Middle Paleoindian	Ch	Negative
**91**	NC186	Durham/NC	Clovis	Early Paleoindian	Rhy	Negative
**92**	NC187	Randolph/NC	Clovis	Early Paleoindian	Rhy	Negative
**95**	VS2	Kershaw/SC	Clovis	Early Paleoindian	CQ	Negative
**96**	VS3	Kershaw/SC	Clovis	Early Paleoindian	CQ	Negative
**97**	VS4	Lancaster/SC	Clovis	Early Paleoindian	MTV	Negative
**98**	VS5	Lancaster/SC	Clovis	Early Paleoindian	MTV	Negative
**99**	VS6	Lancaster/SC	Clovis	Early Paleoindian	MTV	Negative
**100**	VS7	Lancaster/SC	Clovis	Early Paleoindian	CQ	Negative
**102**	VS9	Kershaw/SC	Clovis	Early Paleoindian	MTV	Negative
**103**	P-97/SC443	McCormick/SC	Redstone	Middle Paleoindian	CPC	Negative
**104**	SC750	Cherokee/SC	Clovis	Early Paleoindian	FBR	Negative
**105**	SC617	Bamburg/SC	Waisted Lanceolate	Middle Paleoindian	CPC	Negative
**107**	JL2	Cumberland/NC	Redstone	Middle Paleoindian	Ch	Negative
**108**	JL3	Charleston/SC	Clovis	Early Paleoindian	CPC	Negative
**110**	LS 5206	Allendale/SC	Haw River	Early Paleoindian	CPC	Negative
**111**	LS 5213	Allendale/SC	Haw River	Early Paleoindian	CPC	Negative
**112**	LS 5205	Allendale/SC	Haw River	Early Paleoindian	CPC	Negative
**113**	LS 5215	Allendale/SC	Haw River	Early Paleoindian	CPC	Negative
**115**	LS 5210	Allendale/SC	Haw River	Early Paleoindian	CPC	Negative
**116**	LS 5202	Allendale/SC	Haw River	Early Paleoindian	CPC	Negative
**118**	LS 5204	Allendale/SC	Haw River	Early Paleoindian	CPC	Negative
**119**	LS 5212	Allendale/SC	Haw River	Early Paleoindian	CPC	Negative

[1]Raw Materials types include Coastal Plain Chert (CPC), Chert (Ch), Metavolcanic (MTV), Rhyolite (Rhy), Flow-Banded Rhyolite (FBR), Aphyric Rhyolite (AR), Plagioclase-Porphyritic Rhyolite (PPR), Quartz (Q), Crystal Quartz, (CQ), Green Vitric Tuff (GVT), Black Vitric Tuff (BVT), Jasper (Jas), and Black Mingo Chert (BMC).

Supplementary Figures

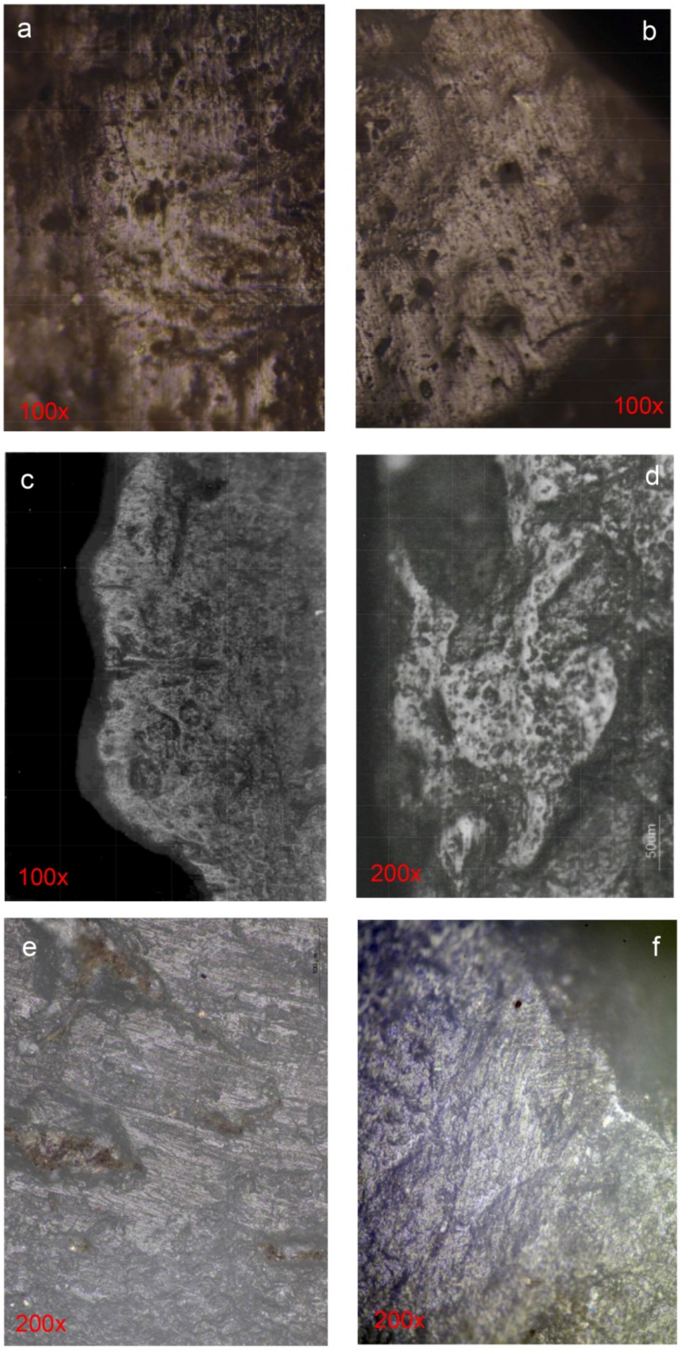

Supplementary Figure 1. Sheath Polish: (a-b) Clovis Knife-120 ST3f-3a; (c) experimental dry hide polish (Kimball[33]:Fig. A38); (d) Neolithic dagger (van Gijn[34]:Fig. 7.11b); (e) Neolithic dagger (van Gijn[35]:Fig. 6.4C); and Dalton point/knife 551.1 (Kimball[36]: Figure 5).

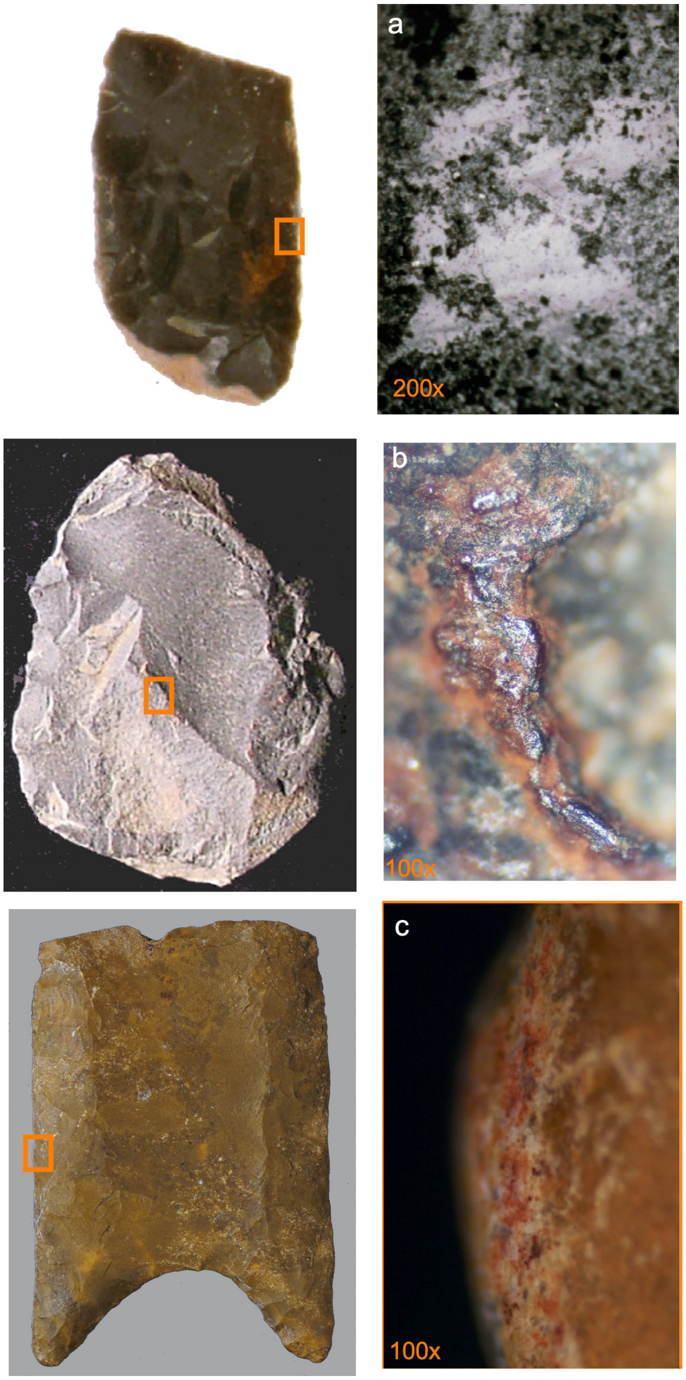

Supplementary Figure 2. Ochred Dry Hide Hafting Polish: (a) Early Archaic bifacial knife I-13 (note patch of ochre) from Main site, KY (Kimball[37]:Figure F-34); (b) Bifacial knife-3402 - 36PE45 PA (Kimball[38]:Plate 24); (e) Clovis 74 (Figure 15 in the main paper).

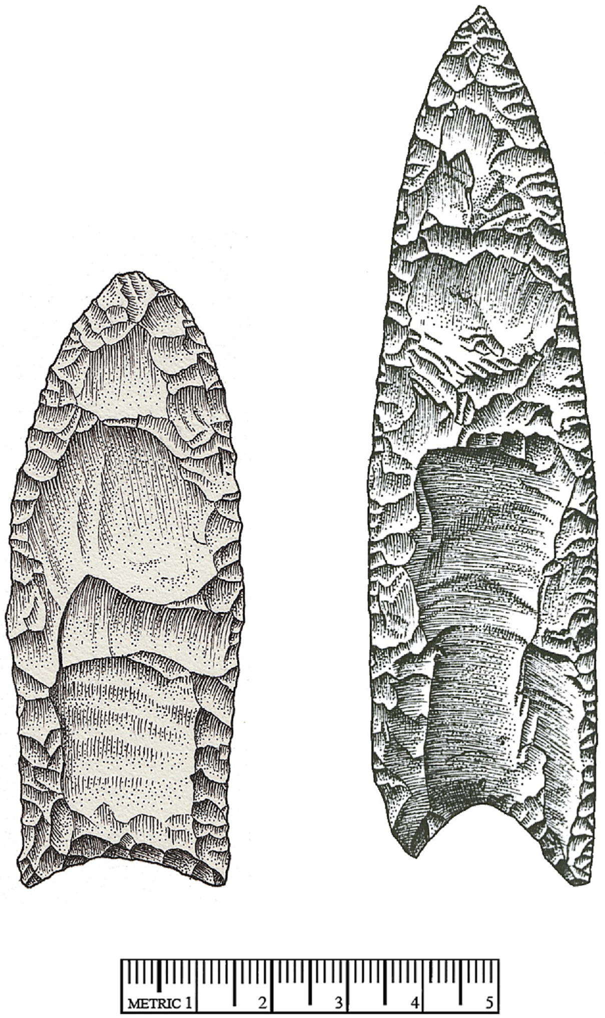

Supplementary Figure 3. Comparison of Clovis (left) and Redstone (right). Drawing by Darby Erd.

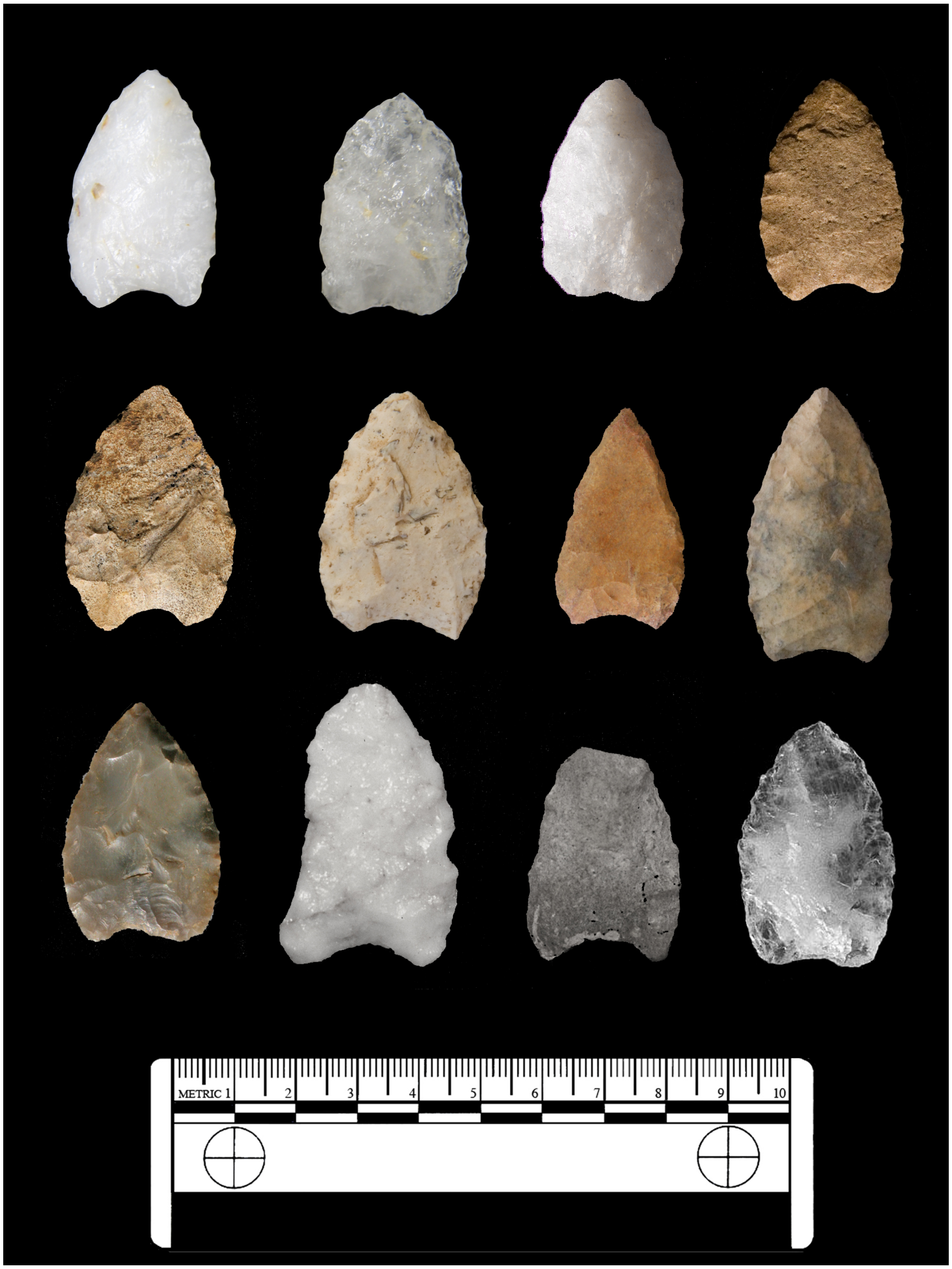

Supplementary Figure 4. Examples of Haw River points (formerly described as "Heart Points") from South Carolina (Charles and Moore [10]).

References

1. Marlar, R. A. Puseman, K. and Linda Scott Cummings, Protein Residue Analysis of Archaeological Materials: Some Comments on Criticisms and Methods. *Southwestern Lore* **61**(2):27-37 (1995).

2. Forgeng, E. Blood Residue Analysis of Seven Crescents from Sonoma County, California. Archaeological Investigations Northwest, Inc. Residue Analysis Report No. 97/399 . Report to the Anthropological Studies Center, Sonoma State University (1998).

3. Loy, T. and James E. J. Dixon Blood Residues on Fluted Points from Eastern Beringea. *American Antiquity* **63**(1):21-46 (1998).

4. Williams, S. B. Blood residue Analysis of a Clovis Point from the Needles, California Resource Area. Archaeological Investigations Northwest, Inc. Residue Analysis Laboratory Letter Report No. 93/144. Prepared for the USDI Bureau of Land Management, Needles, California (1993).

5. Prager, E. M., Wilson, A. C. Lowenstein, J. M. and Sarich V. M. Mammoth Albumin. *Science* 209:287-289 (1980)

6. Dorrill, M., and Whitehead, P. H. The Species Identification of Very Old Human Bloodstains. Forensic Science International   **13**:111-116 (1979).

7. Kind, S. S., and Cleevy R. The Use of Ammoniacal Bloodstain Extracts in ABO Groupings. *Journal of Forensic Sciences* **9**:131-139 (1969).

8. Newman, M. E. The Hidden Evidence from Hidden Cave, Nevada: An Application of Immunological Techniques to the Analysis of Archaeological Materials. Ph.D. Dissertation, Department of Anthropology, University of Toronto, Ontario, Canada (1990).

9. Culliford, B. J. The Examination and Typing of Bloodstains in the Crime Laboratory. National Institution of Law Enforcement and Criminal Justice, U.S. Printing Office, Washington, D.C (1971).

10. Charles, T. and Moore, C. R. *Prehistoric Chipped Stone Tools of South Carolina.* Piedmont Archaeological Studies Trust (P.A.S.T). Piedmont Archaeological Studies Trust Inc., Glendale, South Carolina (2018).

11. Coe, J. L. *The Formative Cultures of the Carolina Piedmont.* Transactions of the American Philosophical Society Vol. 54, Part 5. The American Philosophical Society, Independence Square, Philadelphia (1964).

12. Goodyear, A. C. The Brand Site: A Techno-Functional Study 0/ a Dalton Site in Northeast Arkansas. Arkansas Archaeological Survey, Research Series 7. Fayetteville (1974).

13. Daniel, I. R., Jr. Time, Typology, and Point Traditions in North Carolina Archaeology: Formative Cultures Reconsidered. The University of Alabama Press, Tuscaloosa (2021).

14. Adovasio, J. M. *et al.* No Vestige of a Beginning nor Prospect for an End: Two Decades of Debate on Meadowcroft. In *Ice Age Peoples of North America: Environments, Origins, and Adaptations of the First Americans*, edited by Robson Bonnichsen and Karen. L. Turnmire, pp. 416–431. Oregon State University Press, Corvallis (1999).

15. McAvoy, J. M., and McAvoy, L. D. *Archaeological Investigations of Site 44SX202, Cactus Hill, Sussex County, Virginia*. Research Report Series No. 8. Virginia Department of Historic Resources, Richmond, Virginia (1997).

16. Painter, F. One Man's Trash is Another Man's Treasure: A Study in Discarded Tools and Weapons. *The Chesopiean* **20**, Nos. 5-6, pp.17-37. (Haw River type description on p. 19). Published by The Chesopiean Archaeological Association (1982).

17. Painter, F. Two Basic Paleoindian Lithic Traditions Evolving from a Southeastern Hearth (A Revolutionary Idea). *Archaeology of Eastern North America* **11**:65-79 (1983).

18. Gingerich, J. A. M. and Childress, W. A. Evolving Perspectives on Virginia's Paleoindian Record. In *The American Southeast at the End of the Ice Age*, pp 171-112, edited by D. Shane Miller, Ashley M. Smallwood and Jesse W. Tune. The University of Alabama Press, Tuscaloosa (2022).

19. Whatley, J. S. and Arena, J. W. Jr. An Overview of Georgia Projectile Points and Selected Cutting Tools, Second Edition. The Archaeological Society of South Carolina (2021).

20. Goodyear, A. C., III. , Instrument-Assisted Fluting as a Technochronological Marker Among North American Paleoindian Points. *Current Research in the Pleistocene* **27**:86–88 (2010).

21. Cambron, J. W., and Hulse, D. C. *Handbook of Alabama Archaeology, Point Types Part 1* (1964).

22. Smallwood, A. M., and T. A. Jennings, (editors) Clovis: On the Edge of a New Understanding. Texas A&M University Press, College Station (2015).

23. Morrow, J. E. Clovis-era Lithic Technology in the Midcontinent by Juliet E. Morrow. In *Clovis*: *On the Edge of a New Understanding*, edited by Ashley M. Smallwood and Thomas A. Jennings, pp. 83-108. Texas A&M University Press, College Station (2015).

24. Brooster, J. B. and Norton, M. R. The Tennessee Fluted Point Survey PIDBA database (2005).

25. Goodyear, A. C., III. Recognizing the Redstone Fluted Point in the South Carolina Paleoindian Point Database. *Current Research in the Pleistocene* **23**:100–103 (2006).

26. McAvoy, J. M., and McAvoy, L. D. Cactus Hill and Other Excavated Sites. Nottoway River Survey *Research Report* No. 5 (2005).

27. Tune, J. W. The Clovis-Cumberland-Dalton Succession: Settling into the Midsouth United States during the Pleistocene to Holocene Transition. *PaleoAmerica* 2: 261–273 (2016).

28. Bullen, R. A Guide to the Identification of Florida Projectile points, Revised edition. Kendall, Books T.M., Gainesville, Florida (1975).

29. Meeks, S. C., and Anderson, D. G. Evaluating the Effect of the Younger Dryas on Human Population Histories in the Southeastern United States. In *Hunter-Gatherer Behavior Human Response During The Younger Dryas*, edited by Metin I. Eren, pp. 111–138. Left Coast Press, Walnut Creek, California (2015).

30. Dunbar, J. S. *Paleoindian Societies of the Coastal Southeast.* 1st ed., University Press of Florida. *JSTOR*, https://doi.org/10.2307/j.ctvx06zz3 (2016).

31. DeJarnette, D. L., Kurjack, E. and Cambron, J. W. Stanfield-Worley Bluff Shelter Excavations. *Journal of Alabama Archaeology* **8**, Nos. 1 and 2. Alabama Archaeological Society, University Alabama (1962).

32. Soday, Frank J. and Cambron, J. W. A survey of Paleoindian sites and artifacts in the Tennessee River Valley (1959).

33. Kimball, L. R. *Planning and functional variability in the Upper Palaeolithic: Microwear analysis of Upper Perigordian tools from Le Flageolet I (Dordogne).* Ph.D. dissertation. Northwestern University (1989).

34. van Gijn, A.L. *Flint in focus: Lithic biographies in the Neolithic and Bronze Age*. Sidestone Press, Leiden (2010).

35. van Gijn, A. L. The cultural biography of the Scandinavian daggers in the northern Netherlands. In *Flint daggers in prehistoric Europe,* edited by C.J. Frieman and B.V. Eriksen, pp. 76-82. Oxbow Books, Oxford (2015).

36. Kimball, L. R. *Microwear analysis of lithic tools from 11PK1771, Pike County, Illinois*. Technical Report submitted to URS Corporation, Burlington, New Jersey (2009).

37. Kimball, L. R. Microwear analysis of Archaic and Early Woodland tools from the Main site (15BL35), Kentucky. In *Upper Cumberland Archaic and Woodland Period at the Main site (15BL35), Bell County, Kentucky*, by S.D. Creasman, Appendix F. Report by Cultural Resource Analysts, Inc. to the KDOT and USCOE (1994b).

38. Kimball, L.R. Microwear analysis of Archaic tools from 36Pe45 Pennsylvania. In *Archaeological Investigations, Route 11/15 Improvements (SR0011, Section 005), Juniata and Perry Counties, Pennsylvania*, by P.E. Miller, N. Sheehan, and J.T. Marine. Report submitted to the Pennsylvania Department of Transportation, Engineering District 8-0, Harrisburg, PA (2005).
